# Moderators of inflammation-related depression: a prospective study of breast cancer survivors

**DOI:** 10.1038/s41398-021-01744-6

**Published:** 2021-12-06

**Authors:** Andrew W. Manigault, Patricia A. Ganz, Michael R. Irwin, Steve W. Cole, Kate R. Kuhlman, Julienne E. Bower

**Affiliations:** 1 Department of Psychology, UCLA, Los Angeles, CA USA; 2 David Geffen School of Medicine, UCLA, Los Angeles, CA USA; 3 Health Policy and Management, UCLA Fielding School of Public Health, Los Angeles, CA USA; 4 Department of Psychiatry and Biobehavioral Sciences, UCLA, Los Angeles, CA USA; 5 Cousins Center for Psychoneuroimmunology, Semel Institute for Neuroscience and Human Behavior, UCLA, Los Angeles, CA USA; 6 Department of Psychological Science, UCI, Irvine, CA USA

**Keywords:** Physiology, Depression

## Abstract

Inflammation has been shown to predict depression, but sensitivity to inflammation varies across individuals. Experimental studies administering potent pro-inflammatory agents have begun to characterize this sensitivity. However, risk factors for inflammation-associated depression in naturalistic contexts have not been determined. The present study examined key psychological and behavioral risk factors (state anxiety, perceived stress, negative affect, disturbed sleep, and childhood adversity) as potential moderators of the relationship between inflammation and depressive symptoms in a prospective longitudinal study of breast cancer survivors. Women with early stage breast cancer were recruited after completing primary cancer treatment (*n*_final_ = 161). Depressive symptoms, inflammatory markers (CRP, IL-6, and sTNF-RII), and key risk factors were assessed post treatment (T1), at 6 and 12-month follow-ups (T2 and T3), and during a final follow-up (TF) 3−6 years after T1; childhood adversity was measured only at T3. Inflammatory markers were combined into a single inflammatory index prior to analyses. Women who reported higher levels of state anxiety, perceived stress, negative affect, and/or sleep disturbance at T1 (post-treatment) exhibited higher depressive symptoms at times when inflammation was higher than typical (interaction βs ranged from .06 to .08; all *p*s < .014). Results demonstrate the relevance of these risk factors for understanding inflammation-associated depression in a clinical context and could inform targeted strategies for prevention and treatment among at-risk populations.

## Introduction

Depression is a prevalent [1] and debilitating disorder, which ranks among the leading causes of worldwide disability [2]. Notably, depression is linked to inflammation [3–6], such that inflammatory stimulation causes sickness behaviors that include depressive symptoms [7–9], inflammatory markers are elevated among depressed individuals [6], and inflammation precedes depressive symptoms in large longitudinal studies [10] (although these prospective effects are small and likely bidirectional [11]). However, there is growing recognition that inflammation is not an “equal opportunity” inducer of depressive symptoms [12, 13]. For example, only a subset of individuals develops major depressive disorder following interferon-alpha (IFN-α) treatment—a potent and chronic pro-inflammatory agent [14]. To explain this variability, emerging theories have adopted a “two hit” view of depression where the combination of inflammation and select vulnerability factors is hypothesized to predict depression [5, 15, 16]. Experimental studies that induce depressive symptoms using potent pro-inflammatory agents (e.g., IFN-α, endotoxin) have begun to elucidate potential risk factors for inflammation-related depression. Notably, endotoxin studies find that a range of risk factors, including female sex [17], baseline activity of inflammatory, beta-adrenergic, and glucocorticoid signaling transcription factors [18], sleep disturbance [19], perceived stress, trait sensitivity to social disconnection, and pre-existing symptoms of depression and anxiety [16], all increase severity of depressive symptoms following endotoxin administration. Similarly, IFN-α studies find that individuals who develop major depressive disorder during the course of treatment exhibit higher inflammatory reactivity [20–24], greater glucocorticoid reactivity [25, 26], and higher levels of pre-treatment neuroticism, depression and sleep disturbance (and trend towards increased anxiety) [27–29]. Other work has found that early life stress increases susceptibility to depressive symptoms during the acute response to vaccine administration [30]. Of note, the endotoxin and IFN-α models both produce notably strong inflammatory responses (e.g., IL-6 increases ranging from 46 to 190 pg/mL), and in the case of IFN-α, target specific populations (i.e., hepatitis C/malignant melanoma patients) [8, 9, 25].

By contrast, real-life fluctuations in inflammation are typically much lower in magnitude, but could still contribute to depression risk [10]. To date, only a few studies have examined moderators of inflammation-related depression in naturalistic contexts. One study found a potentiating effect of childhood stress exposure in adolescents [31], where greater childhood stress was associated with a stronger coupling of inflammation and depression over a 2.5-year period. Another study examined these effects among women facing the stress of breast cancer diagnosis and treatment [32], and found that cancer-related stress strengthened the association between inflammation and depressive symptoms over a 2-year period. These studies provide initial real-world evidence for a role of stress as a risk factor for inflammation-related depression [5]. Nevertheless, other notable risk factors suspected of interacting with inflammation warrant evaluation to determine whether they might be useful for risk assessment and potentially, targeted intervention.

The present report used an existing prospective, longitudinal, cohort study of breast cancer survivors [33–36] to examine five key risk factors—anxiety, negative affect, perceived stress, sleep disturbance, and childhood adversity—as moderators of the association between inflammation and depressive symptoms. These risk factors were chosen because they are all established independent predictors of depression onset [28, 37–41] that were found to predict inflammation-related depression in experimental studies [16, 19, 27–30]. Understanding risk factors for depression in breast cancer survivors is particularly important because depression prevalence is increased in this population [42] and is known to impact treatment adherence [43], health care utilization [44], and mortality [45]. Moreover, the physical and psychological stressors of breast cancer diagnosis and treatment may activate inflammatory pathways, leading to elevated inflammation in survivors [46, 47]. In this trial, women with early-stage breast cancer were recruited after completing primary cancer treatment and completed 4 assessments over a 3 to 6-year period [33–36]. Based on results from experimental studies [16, 19, 27–29], we hypothesized that women who reported higher post-treatment anxiety, negative affect, perceived stress, sleep disturbance, and childhood adversity would display a stronger association between inflammation and depressive symptoms at any given time. We focused on post-treatment risk factors because this is a potentially stressful “reentry period” for breast cancer survivors who often feel pressured to reengage with responsibilities (e.g., child care, work) while losing access to treatment-related care resources [48].

## Methods and materials

### Participants

This study is a secondary analysis of data collected as part of the Mind Body Study (MBS)—a prospective, longitudinal, cohort study of breast cancer survivors designed to evaluate the impact of endocrine therapy on cognitive function [33–36]. Eligibility criteria for the parent study reflected the focus on cancer-related cognitive function and adjuvant endocrine therapy treatment for breast cancer. Inclusion criteria were: (1) aged 21−65, (2) newly diagnosed with early stage breast cancer (stage 0-IIIA), (3) had completed primary cancer treatment (surgery, radiation, and/or chemotherapy) within the last 3 months, (4) had yet to start endocrine therapy, if indicated, (5) were proficient in English, and (6) were available for in-person follow-up assessments. Participants were considered ineligible if they reported any of the following: (1) current diagnosis of uncontrolled major affective disorder, (2) current/past psychotic-spectrum disorder, (3) substance use/dependence, (4) daily tobacco/alcohol use, (5) prior cancer diagnosis/chemotherapy treatment, (6) insulin-dependent diabetes or auto-immune disease, (7) chronic use of oral steroids or hormone therapy other than vaginal estrogen, (8) uncontrolled allergic reaction/asthma, (9) prior brain irradiation/surgery, (10) past/current diagnosis with a central nervous system disorder or a condition impacting cognitive functioning, (11) epilepsy, dementia, or learning disability. A total of 191 women were enrolled in the parent study; sample size was determined based on the objectives of the parent study.

**Fig. 1:**
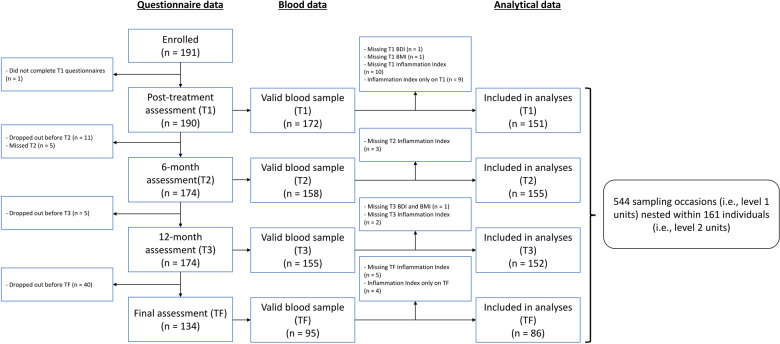
Study flow chart. A total of 161 participant contributed 544 (151 + 155 + 152 + 86) sampling occasions across the full study period. It should be noted that some participants contributed valid level 1 observations at some time points but not others, and were still in analyses if they contributed at least 2 valid sampling occasions (e.g., missing T1 inflammation and depression data but valid data at T2 and T3). For this reason, the total number of level 2 units (161 participants) is greater than the total number of level 1 units (sampling occasions) at any given time point of the study (151, 155, 152, and 86).

As the focus of this study was on the role of inflammation and depression, a subsample of MBS patients was used for the purposes of this investigation. Participants were included in this subsample if they provided valid data for depressive symptoms *and* inflammatory markers on at least 2 out of the 4 study time points (a necessity for valid within-person centering). Of note, data for all three inflammatory markers were required for computing the inflammation index. As shown on Fig. 1, 161 women met these criteria and were included in the final sample. Of these 161 women, 160 had valid values for T1 anxiety, perceived stress, and negative affect, whereas 157 had valid T1 sleep disturbance values and 155 had valid T3 childhood adversity data. Accordingly, analytical samples for the present study included between 155 and 161 women (depending on whether/which risk factor was included in analyses).

### Procedure

As previously described [33–36], study participants were recruited between 2007 and 2010 from the Los Angeles area using cancer registries from regional hospitals. MBS participants completed up to four assessments that involved completion of questionnaires and collection of blood samples. The first assessment (T1) took place after the end of primary breast cancer treatment and before beginning endocrine therapy [34]. Next, participants completed assessments scheduled 6 (T2) and 12 (T3) months after T1 [33, 36]. Between March 2013 and July 2014, a “final” assessment (TF) was completed by a subsample of participants; the delay between T1 and TF ranged from 3 to 6 years (mean = 4.3) [35]. Study procedures were pre-approved by the UCLA Institutional Review Board; all participants provided written informed consent.

### Measures

Blood was collected during morning visits (before 11 AM) that were preceded by an overnight fast. Participants were asked about recent illness or infection and were rescheduled if either was endorsed. Blood samples were collected via venipuncture during each in-person assessment (T1-TF), and were processed and assayed as previously described [46]. Briefly, blood samples were collected in EDTA tubes, placed on ice, then centrifuged for plasma acquisition, and stored at −80 °C until assayed in batch. Enzyme-linked immunosorbent assays were used to quantify plasma levels of C-reactive protein (CRP; Immundiagnostik, ALPCO Immunoassays, Salem, NH; 0.2 mg/L lower limit), interleukin-6 (IL-6; R&D Systems, Minneapolis, MN; 0.2 pg/ml lower limit), and soluble tumor necrosis factor receptor type II (sTNF-RII; R&D Systems, Minneapolis, MN; 234 pg/ml lower limit) according to the manufacturer’s protocol. All samples were run in duplicate; the intra- and inter-assay precision of all tests were less than or equal to 10%. Values of CRP, IL-6, and sTNF-RII were combined into a single inflammatory index to provide an integrated assessment of inflammation and thereby generate estimates of overall inflammatory biology [49] as well as avoid competing predictors and multiple hypothesis testing. A valid value for each marker (i.e., CRP, IL-6, and sTNF-RII) was necessary to compute the inflammation index. The inflammation index was computed as follows: (1) each marker was natural log-transformed to address skewness, (2) log-transformed values were then standardized, and (3) the resulting z-scores were summed.

Participants were mailed a questionnaire packet (and asked to complete it) prior to in-person visits. These questionnaire packets included measures of depressive symptoms, state anxiety, perceived stress, negative affect, and sleep disturbance. During T3, participants also completed a measure of childhood adversity. Depressive symptoms were measured using the 21-item Beck Depression Inventory-II (BDI-II), which assesses affective, cognitive, and vegetative symptoms of depression over the past two weeks [50]. State anxiety was assessed using the 20-item State Trait Anxiety Inventory (STAI) which queries present moment feelings of anxiety [51]. Perceptions of stress over the last month were assessed using the 10-item Perceived Stress Scale (PSS) [52]. The 10-item negative affect subscale of the Positive Affect Negative Affect Scale (PANAS) [53] was used to index negative affect over the past week. The 19-item Pittsburgh Sleep Quality Inventory (PSQI) [54] was used to measure sleep disturbance over the past month. Childhood adversity was measured using the 13-item Risky Families (RF) questionnaire [55]. Across assessments, each of these scales exhibited excellent reliability (Cronbach’s α ranging from .89 to .91), with the exception of the PSQI which showed moderate reliability (Cronbach’s α ranging from .60 to .71). The RF scale also exhibits excellent reliability (Cronbach’s α = .89).

Demographic and medical information were obtained via self-report at T1 or from medical record abstraction. Physical measurements (i.e., height and weight) were obtained during each visit. Among the measures collected were age, race, BMI, cancer stage, surgery type, and receipt of chemotherapy, radiation therapy, and endocrine therapy. These variables were used as covariates, consistent with prior work [32, 56].

### Analytic plan

Tests of primary hypotheses relied on 2-level robust multilevel models carried out using the robustlmm package [57] on R 4.0.3 [58], where repeated assessments of inflammation and depressive symptoms (level 1) were nested within individuals (level 2). To model how inflammation fluctuates around a person’s typical levels, the inflammatory index was centered relative to persons, consistent with prior work [32, 59]. Accordingly, positive person-centered scores indicate that inflammation levels at a particular visit exceeded average inflammation levels for that individual. Person-centered inflammation was entered as a level 1 fixed effect. Risk factors at T1 (i.e., state anxiety, perceived stress, negative affect, and sleep disturbance) and childhood adversity at T3 were each entered in separate models as level 2 fixed effects. Test of primary hypotheses examined the 2-way interaction of person-centered inflammation and each risk factor variable and thereby test whether the relationship between inflammation and depression at any given assessment is moderated by the T1 value of that risk factor (or T3 for childhood adversity). Follow up tests of simple slopes contrasted the association between person-centered inflammation and depressive symptoms at low (−1 SD), average (mean), and high (+1 SD) levels of risk factor variables.

Six sets of secondary analyses were carried out. First, primary analyses were repeated using person-centered (to quantify within-person variability) or grand mean centered (to quantify between-person variability) versions of the anxiety, perceived stress, negative affect, and sleep disturbance moderator variables. These analyses included repeated assessments of the risk factors to determine whether the moderating effect of these variables was driven by T1 levels (the focus of primary analyses), within-person variability in risk factors, or average levels of these risk factors across the study period. Second, we tested the between-subject association between depression and inflammation, whether this association was moderated by risk factors, and whether primary analyses remained significant when controlling for these interactions. Third, all five risk factors were tested in a single model to evaluate the relative contribution of each risk factor to the association between inflammation and depressive symptoms (i.e., were moderating effects of each risk factor independent of one another?). Fourth, significant primary analyses were repeated for each individual inflammatory marker (instead of the inflammatory index) to examine if effects of the combined index were specific to one or multiple inflammatory markers. Fifth, we tested three-way interactions of age by inflammation by key risk factors to examine if primary results were comparable across breast cancer survivors of varied ages. Sixth, significant primary analyses were repeated while excluding BDI items that pertain to anxiety and sleep.

To estimate effect sizes, standardized regression coefficients were computed by standardizing criterion and continuous predictor variables [60]. Final models controlled for age, BMI, race (White v. non-White), receipt of chemotherapy, radiation therapy, endocrine therapy, cancer stage (stage 0−1 v. stage 2−3), and surgery type (lumpectomy v. mastectomy), such that continuous covariates were mean centered and categorial covariates were centered on the most frequent category. Excluding covariate measures from models did not change the interpretation of the results. Family-wise Type I error rates were reduced (to α = .05) using the Holm’s procedure [61] (i.e., family of tests were evaluated using rank-ordered *p*-value thresholds). Plots were created using the sjPlot R package [62].

## Results

### Sample characteristics

As shown in Table 1, study participants were primarily college-educated, middle-aged, white women diagnosed with stage 0 or 1 breast cancer. The majority were treated with radiation therapy and endocrine therapy; approximately half were treated with chemotherapy. As indicated in Fig. 1, the women included in these analyses are a subset of the 191 women originally enrolled in MBS. Participants included (*n* = 161) v. excluded (*n* = 30) from the analytic sample did not differ on the basis of age, education, race, marital status, income, cancer stage, receipt of chemotherapy, or receipt of radiation therapy (all *p*s > 0.05). However, participants included in analytic sample were significantly more likely to have received endocrine therapy than excluded participants (*X*^*2*^(1, *N* = 191) = 7.6, *p* = 0.006, OR = 3.24). Comparison of sample characteristics by primary study variables is presented in supplemental materials.

**Table 1:** Demographics and clinical characteristics of the sample.

Variable	*N*	%	Mean	(SD)
Age (years)	161		51.61	(8.10)
Education				
Less than college	32	19.9%		
College degree	50	31.1%		
Post-graduate degree	79	49.1%		
Race/ethnicity				
White/Caucasian	126	78.3%		
Hispanic/Latino	17	10.6%		
Black/African American	5	3.1%		
Asian	8	5.0%		
Other	5	3.1%		
Married				
Married or living as married	107	66.9%		
Divorced, separated, widowed, or never married	53	33.1%		
Annual household income				
< $100,000	58	36.9%		
> $100,000	99	63.1%		
Breast cancer stage				
0 or 1	94	58.4%		
2 or 3	67	41.6%		
Surgery type				
Lumpectomy	108	67.1%		
Mastectomy	53	32.9%		
Received chemotherapy	88	54.7%		
Received radiation therapy	121	75.2%		
Received endocrine therapy	119	73.9%		

As shown in Table 2, on average, study participants reported T1 symptoms below the clinical cutoff scores for depression (BDI-II < 13) [50] and anxiety (SAI < 40) [63]. By contrast, average levels of sleep disturbance exceeded the clinical cutoff of 5 on the PSQI [54], suggesting poor sleep. Similarly, average perceived stress levels were elevated relative to age-matched norms (45−54 year olds: *M* = 12.6) [52].

**Table 2:** Post-treatment (T1) descriptive statistics and zero-order correlations.

Variable	*M*	SD	1	2	3	4	5	6	7	8	9
1. Depressive symptoms (BDI-II)	8.69	6.92									
2. State anxiety (SAI)	35.00	8.47	0.74**								
3. Perceived stress (PSS)	13.96	6.75	0.71**	0.74**							
4. Negative affect (PANAS)	16.28	5.86	0.71**	0.75**	0.71**						
5. Sleep disturbance (PSQI)	7.54	3.49	0.47**	0.31**	0.36**	0.33**					
6. Childhood adversity (RF)^a^	27.83	10.44	0.27**	0.26**	0.23**	0.23**	0.05				
7. Composite inflammatory index	0.53	2.22	0.16	0.13	0.08	0.01	0.14	0.19*			
8. CRP (mg/L)	2.14	2.83	0.07	0.07	0.06	−0.05	0.12	−0.00	0.59**		
9. IL-6 (pg/mL)	1.65	1.03	0.09	0.10	0.05	0.05	0.12	0.10	0.71**	0.46**	
10. sTNF-RII (pg/mL)	2289.01	623.57	0.07	0.04	−0.01	−0.07	0.02	0.15	0.66**	0.08	0.18*

*Note*. M = mean; SD = standard deviation; *p < .05; **p < .01. *BDI-II* Beck Depression Inventory II; *SAI* State Anxiety Questionnaire; *PSS* Perceived Stress Scale; *PANAS* Positive Affect Negative Affect Scale; *PSQI* Pittsburg Sleep Quality Inventory; *RF* Risky Families questionnaire; *CRP* C-reactive Protein; *IL-6* Interleukin 6; *sTNF-RII* soluble tumor necrosis factor receptor type II.

^a^ All variables listed in this table were collected at T1, with the exception of the Risky Families questionnaire which was collected at T3.

### Stability and temporal effects

The Intra-Class Correlation (ICC) for depressive symptoms scores was .69; the ICC coefficient decomposes total variability into its between- and within-subject components, and thus suggests that 69% of the total variance in depressive symptoms could be attributed to differences between individuals. Similar ICC estimates were obtained for anxiety (ICC = 0.67), perceived stress (ICC = 0.72), negative affect (ICC = 0.61), sleep disturbance (ICC = 0.58), IL-6 (ICC = 0.64), sTNF-RII (ICC = 0.73), and CRP (ICC = 0.84). See Supplementary Table S1 for descriptive statistics of these variables at each study timepoint.

Levels of depressive symptoms showed a non-significant association with time in months since T1 (*b* = −0.012, SE = 0.008, *β* = −0.03, *t*(442) = 1.5, *p* = 0.13), suggesting that, across the full sample, depressive symptoms remained relatively stable across time. Similarly, levels of the inflammatory index showed a non-significant association with time in months since T1 (*b* = −0.004, SE = 0.003, *β* = −0.04, *t*(399) = 1.34, *p* = 0.18), suggesting that, across the full sample, inflammation remained relatively stable across time.

### Associations between depressive symptoms, risk factors, and markers of inflammation

At the initial (T1) assessment, depressive symptoms, state anxiety, perceived stress, negative affect, and sleep disturbance were all positively correlated (*r* range: 0.31−0.75; see Table 2). Childhood adversity was positively correlated with T1 depressive symptoms, state anxiety, perceived stress, negative affect (*r* range: 0.23−0.27), but not significantly correlated with T1 sleep disturbance (*r* = 0.05). T1 levels of the inflammatory index (and each inflammatory marker) were not significantly correlated with T1 risk factors (*r* range: −0.07−0.16), but were positively correlated with childhood adversity (*r* = 0.19). T1 inflammatory markers were all strongly correlated with the inflammatory index at T1 (*r* range: 0.59−0.71), but less consistently associated with one another. More specifically, T1 IL-6 was positively correlated with T1 CRP (*r* = 0.46), and sTNF-RII (*r* = 0.18), but the association between T1 CRP and T1 sTNF-RII was non-significant (*r* = 0.08).

Analyses were also conducted to evaluate associations between T1 risk factors and average levels of depression and inflammation across the full study period. Examining average depressive symptoms across time revealed that post-treatment (T1) levels of state anxiety (*b* = 0.48, SE = 0.04, *β* = 0.57, *t*(153) = 11.97, *p* < 0.001), perceived stress (*b* = .53, SE = 0.05, *β* = 0.51, *t*(152) = 9.91, *p* < 0.001), negative affect (*b* = 0.67, SE = 0.06, *β* = 0.56, *t*(153) = 10.90, *p* < 0.001) and sleep disturbance (*b* = 0.62, SE = 0.12, *β* = 0.31, *t*(146) = 5.00, *p* < 0.001) were all positively associated with average depressive symptoms across the full study period. By contrast, examining average inflammation across time revealed that post-treatment (T1) levels of state anxiety (*b* = 0.03, SE = 0.02, *β* = 0.10, *t*(150) = 1.74, *p* = 0.08), perceived stress (*b* = 0.02, SE = 0.02, *β* = 0.05, *t*(150) = 0.93, *p* = 0.35), negative affect (*b* = 0.01, SE = 0.02, *β* = 0.02, *t*(150) = 0.42, *p* = 0.67) and sleep disturbance (*b* = 0.04, SE = 0.04, *β* = 0.06, *t*(144) = 0.97, *p* = 0.33) were not significantly associated with average scores on the inflammation index across the full study period.

Finally, tests of within-person associations revealed that person-centered inflammation was not significantly associated with depressive symptoms (*b* = 0.15, SE = 0.13, *β* = 0.02, *t*(392) = 1.10, *p* = 0.27), suggesting that, depressive symptoms did not significantly increase at times when inflammation was higher than usual.

### Moderators of the link between depressive symptoms and person-centered inflammation

**Fig. 2:**
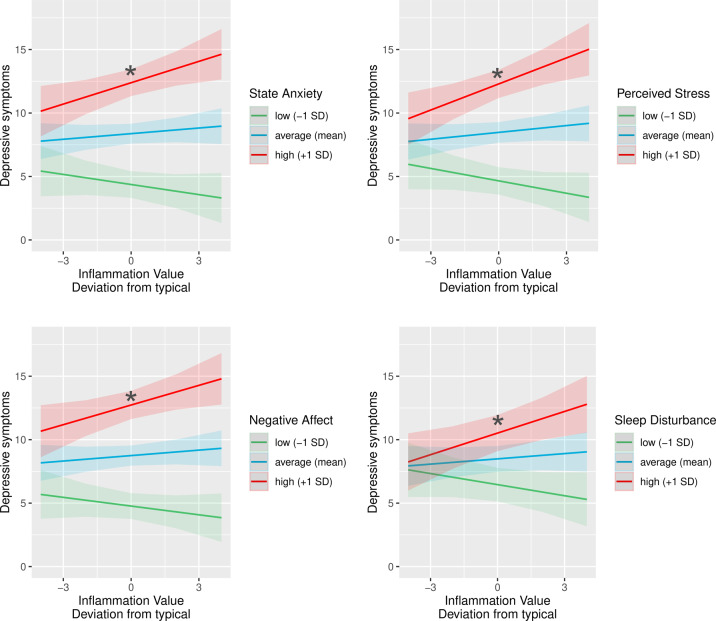
Predicted depressive symptom scores (at any given time) as a function of person-centered inflammation (at any given time) and T1 psychological risk factors (state anxiety, perceived stress, negative affect, and sleep disturbance). Inflammation (as indexed by a composite measure of CRP, IL-6, and sTNF-RII) was positively associated with depressive symptoms at any given time among women who reported high levels of T1 anxiety, perceived stress, negative affect, and sleep disturbance. Predicted depressive symptom scores were adjusted for age, BMI, race, surgery, cancer stage, and receipt of chemotherapy, radiation therapy, and endocrine therapy. Shaded areas depict confidence intervals of simple slopes. The asterisk symbols (*) index statistically significant simple slopes (*p* < 0.05).

Primary analyses tested the hypothesis that the association between inflammation and depressive symptoms at any given time would be stronger among individuals who report higher post-treatment (T1) levels of state anxiety, perceived stress, negative affect, sleep disturbance, and higher childhood adversity. Results supported this hypothesis (see Table 3), demonstrating that the association between person-centered inflammation and depressive symptoms was moderated by post-treatment levels of state anxiety (*b* = 0.05, SE = 0.02, *β* = 0.06, *t*(382) = 2.76, *p* = 0.006), perceived stress (*b* = 0.07, SE = 0.02, *β* = 0.08, *t*(382) = 3.40, *p* < 0.001), negative affect (*b* = 0.06, SE = 0.02, *β* = 0.06, *t*(387) = 2.46, *p* = 0.014) and sleep disturbance (*b* = 0.11, SE = 0.04, *β* = 0.06, *t*(376) = 2.81, *p* = 0.005). By contrast, childhood adversity did not moderate the association between person-centered inflammation and depressive symptoms (*b* = 0.001, SE = 0.01, *β* = 0.001, *t*(374) = 0.08, *p* = 0.94). As shown in Fig. 2 and Table S2, tests of simple slopes revealed that inflammation was positively associated with depressive symptoms among women who reported high (+1 SD) levels of state anxiety (*b* = 0.57, SE = 0.20, *β* = 0.09, *t*(387) = 2.91, *p* = 0.004), perceived stress (*b* = 0.70, SE = 0.20, *β* = 0.11, *t*(387) = 3.51, *p* < 0.001), negative affect (*b* = 0.53, SE = 0.20, *β* = 0.09, *t*(392) = 2.70, *p* = 0.007) and sleep disturbance (*b* = 0.54, SE = 0.19, *β* = 0.09, *t*(383) = 2.78, *p* = 0.006). By contrast, the association between inflammation and depressive symptoms was non-significant among women who reported low (−1 SD) and average (mean) levels of state anxiety (*b* = −0.21, SE = 0.20, *β* = −0.03, *t*(383) = 1.05, *p* = 0.29, and *b* = 0.18, SE = 0.14, *β* = 0.03, *t*(388) = 1.33, *p* = 0.19, respectively), perceived stress (*b* = −0.25, SE = 0.19, *β* = −0.04, *t*(383) = 1.34, *p* = 0.18, and *b* = 0.22, SE = 0.14, *β* = 0.04, *t*(389) = 1.65, *p* = 0.09, respectively), negative affect (*b* = −0.15, SE = 0.19, *β* = −0.02, *t*(384) = 0.79, *p* = 0.43, and *b* = 0.19, SE = 0.14, *β* = 0.03, *t*(390) = 1.40, *p* = 0.16, respectively) and sleep disturbance (*b* = −0.23, SE = 0.19, *β* = −0.04, *t*(375) = 1.20, *p* = 0.23, and *b* = 0.16, SE = 0.13, *β* = 0.03, *t*(382) = 1.16, *p* = 0.25, respectively). Performing Holm’s adjustment [61] did not alter these results. Controlling for psychotropic medication use did not influence results.

**Table 3:** Standardized coefficients for multilevel models predicting depressive symptoms as a function of the composite inflammatory index and key risk factors (anxiety, stress, negative affect and sleep disturbance).

	Model 1		Model 2		Model 3		Model 4		Model 5	
	*β*	(SE)	*β*	(SE)	*β*	(SE)	*β*	(SE)	*β*	(SE)
Inflammation	.02	(.02)	.03	(.02)	.04	(.02)	.03	(.02)	.03	(.02)
State anxiety			.58*	(.05)						
Inflammation × state anxiety			.06*	(.02)						
Perceived stress					.52*	(.05)				
Inflammation × perceived stress					.08*	(.02)				
Negative affect							.57*	(.05)		
Inflammation × negative affect							.06*	(.02)		
Sleep disturbance									.30*	(.06)
Inflammation × sleep disturbance									.06*	(.02)

Notes: *p < 0.05; Inflammation = scores on the composite inflammatory index. All models shown controlled for age, BMI, race, receipt of chemotherapy, radiation therapy, and endocrine therapy, cancer stage, and surgery type.

### Secondary analyses

Secondary analyses (fully reported in supplemental materials) revealed that (1) within-person variability in negative affect was associated with a stronger coupling of inflammation and depressive symptoms at any given assessment, whereas moderating effects of anxiety, perceived stress and sleep disturbance were limited to the initial (T1) assessment; (2) the between-person association between inflammation and depression was non-significant, was not moderated by risk factors, and controlling for the between-subject inflammation-depression association did not alter primary results of the study; (3) no single risk factor predicted vulnerability for inflammation-associated depressive symptoms above and beyond all others; (4) examining individual cytokines produced varying effects dependent on the marker and the risk factor tested (see Table S3 and Fig. S1); (5) primary results were qualified by interactions with age (see Fig. S2), such that two-way interactions of person-centered inflammation by risk factors (predicting depressive symptoms at any given time) were significant in women of average age (~51.6 years) or younger (~43.5 years), but non-significant in older (~59.7 years) women; and (6) repeating primary analyses while excluding depression questionnaire items that overlap with anxiety and sleep disturbance did not influence results. Supplemental materials also include comparisons of demographics and disease/treatment-related variables by depressive symptoms, inflammation, and risk factors (see Tables S4–S9).

## Discussion

Inflammation has been shown to predict depression, but there is variability in sensitivity to inflammation, and risk factors for inflammation-associated depression have not been identified in clinical samples experiencing real-life inflammatory fluctuation. The present study, therefore, used a prospective, longitudinal, cohort study of breast cancer survivors to examine five risk factors suspected of interacting with inflammation to predict depression risk. As hypothesized, we found that women who reported higher levels of key risk factors at the end of cancer treatment were more vulnerable to inflammation-associated depressive symptoms over the subsequent years. Specifically, those with higher levels of state anxiety, perceived stress, negative affect, and sleep disturbance exhibited a larger rise in depressive symptoms at times when inflammation was higher than usual. Of interest, high (+1 SD) levels of state anxiety, perceived stress and sleep disturbance correspond to probable clinical anxiety levels (>40) [64], moderate stress (14−26) [52], and clinically-relevant sleep disturbance (>5) [54]. Finally, childhood adversity did not moderate the link between inflammation and depressive symptoms. The present study serves to characterize vulnerability to inflammation-associated depression in a breast cancer sample, and could inform targeted strategies for prevention and treatment among at-risk populations.

Studies administering an inflammatory challenge (e.g., endotoxin, and IFN-α studies) find that established risk factors for depression are associated with inflammation-induced depressive symptoms [16–19, 25, 27–30, 65]. Such models can interrogate causal relationships between inflammation and depression because they manipulate inflammation, but may generalize poorly to real-life settings where inflammatory fluctuations are typically less pronounced. The present study extends this literature by demonstrating a link between key risk factors and depressive symptoms in an observational study of breast cancer survivors. This is important because the characteristics of the present clinical sample may be particularly relevant to naturally occurring depression. Notably, given that average inflammation levels were within the normal range in the present sample, results suggest that these risk factors remain salient even when fluctuations in inflammation are relatively mild.

The link between inflammation and depression is attributable to a network of neuro-immune communication in which peripheral inflammatory signals interact with the central nervous system, resulting in altered neurotransmission, cognition, and behavior [3, 66]. Pre-clinical evidence indicates that neuro-immune pathways can be moderated by risk factors, including stress, anxiety, and sleep disturbance [67–70]. Sleep-deprived animals exhibit compromised blood brain barrier integrity [70]. Stressed animals exhibit loss of blood brain barrier integrity [67], increased monocyte trafficking to the brain [68], and microglial cell priming [69]. Furthermore, these stress effects are linked with anxiety, such that stressed animals who exhibit greater monocyte migration to the brain and microglial priming also show more anxiety-like behaviors [68, 71, 72]. Altogether, these results link stress, anxiety, and sleep to increased neuro-immune communication or neuroinflammation, and therefore point to several biological mechanisms that could underlie the present results.

The present study also serves to characterize temporal variability in risk for inflammation-associated depression. We chose to focus on post-treatment (T1) levels of anxiety, perceived stress, negative affect, and sleep disturbance because this stage of survivorship typically presents notable challenges [48]. Given that anxiety, perceived stress, negative affect, and sleep disturbance were measured throughout the study, secondary analyses were carried out to examine whether within- or between-person variance in these risk factors could produce comparable interactions. These secondary analyses only revealed one significant interaction, for negative affect, such that the inflammation-depression association was stronger when negative affect was higher than typical for that individual (a within-subject effect). Overall, this pattern of results suggests that assessment of risk factors closer to the time of diagnosis and treatment may be critical for capturing moderating effects. Indeed, we have previously shown that susceptibility to inflammation-related depression was heightened in breast cancer survivors who experienced high stress in the period following diagnosis. [32]

Secondary analyses revealed a unique effect of within-person variability in negative affect, such that women who experienced more negative affect than typical (at a given time) exhibited a stronger coupling of inflammation and depressive symptoms at that time. To the authors’ knowledge, no prior work examines whether within-person variability in depression risk factors predicts sensitivity to inflammation-associated depression. The present effects of negative affect are thereby novel, and may suggest that individuals who exhibit higher affective reactivity in daily life are at greater risk for inflamed depression. Of interest, depression risk is increased in individuals with a tendency to display negative affect in response to daily life stressors [73], and the present results could indicate that inflammation is implicated in this relationship. Of course, replication will be necessary to confirm this hypothesis. Notably, future work in this area may benefit from including neuroticism measures [29] and/or experience sampling methods to better characterize the cause of negative affect reactivity.

Finally, some individual differences may be better indicators of risk for inflammation-associated depression than others. For example, one prior study found that anxiety predicted endotoxin-induced depressive symptom severity above and beyond seven other risk factors [16]. By contrast, the present results did not support this conclusion, as none of our five risk factors predicted inflammation-associated depressive symptoms above and beyond others. Similarly, some inflammatory signaling pathways could be especially relevant for understanding inflammation-associated depression risk. Supporting this view, results were primarily driven by sTNF-RII. More specifically, all interaction tests trended in the same direction when examining CRP, IL-6, and sTNF-RII separately, but were reliably significant only for sTNF-RII. By contrast, IL-6 and CRP both produced non-significant interaction tests. TNF-α signaling may thereby play an important role in producing depressive symptoms, consistent with prior work linking sTNF-RII with cognitive complaints [74] and fatigue [46, 75] in women with breast cancer. In addition, secondary analyses revealed that interactive effects of inflammation with risk factors were driven by women of average age (51.6 years) or younger. By contrast, interactive effects of inflammation with risk factors were non-significant in older women. Accordingly, the present results appear to best characterize vulnerability to inflammation-associated depression in *younger* breast cancer survivors. Younger survivors (<50) are at the highest risk for depression following breast cancer diagnosis and treatment [76], and the present results could serve to characterize this trend.

The strengths and weaknesses of the present study should be acknowledged. The present study included a relatively large sample, examined a clinical population, included up to four repeated assessments over a 3 to 6-year period, and included varied measures of risk factors and inflammation. The present study also excluded clinically depressed individuals during recruitment. Accordingly, results were not driven by individuals with uncontrolled depression at baseline—an important potential confound. At the same time, this may have limited the range of depressive symptoms observed across the study period. On a related note, the focus of this study was on depressive symptoms; thus, relevance for onset of major depressive disorder is an important extension for future research, and would also help to address any conceptual or methodological overlap in risk factors and depressive symptoms. Our assessment schedule was not designed to capture the acute effects of diagnosis and treatment on depressive symptoms and inflammation; instead, there was relative stability in average levels of these measures in the year after treatment completion. The present sample was primarily composed of well-educated White breast cancer survivors which could limit generalizability. Of interest, while unique sources of inflammation have been documented in this population (e.g., cancer treatment [77]), breast cancer survivors tend to exhibit an inflammation-depression association comparable with other populations [78–80] In addition, the content of several risk factor measures overlapped with depressive symptoms scale items. Secondary analyses addressed this issue by removing anxiety and sleep-related BDI-II items from analyses, but future studies could utilize measures of neuroticism to avoid overlap between negative affect and depression. Finally, the present study does not address temporality (e.g., inflammation preceding depressive symptoms on a given sampling occasion). Similarly, the present study was not experimental and thus cannot address causality.

## Conclusions

Experimental evidence suggests that vulnerability to inflamed depression may be indexed by increased anxiety, stress, depressed mood, disturbed sleep, and childhood adversity [16, 19, 25, 27, 28, 30]. The present study extends this literature by evaluating whether these results generalize to a prospective longitudinal study of breast cancer survivors experiencing typical fluctuations in inflammation. We found that women who reported greater levels of state anxiety, perceived stress, negative affect, and sleep disturbance exhibited a larger rise in depressive symptoms at times when inflammation had risen. These results serve to characterize vulnerability to inflammation-associated depression in a breast cancer sample. Finally, the present results may inform targeted strategies for prevention and treatment. For example, assessment of anxiety, perceived stress, disturbed sleep, and/or negative affect could be used to identify women at risk for inflammation-related depression, and therapies that target inflammation might be an effective treatment strategy in these women.

## Supplementary information

Supplemental Materials

## References

[1] Kessler RC, Berglund P, Demler O, Jin R, Merikangas KR, Walters EE (2005). Lifetime prevalence and age-of-onset distributions of DSM-IV disorders in the National Comorbidity Survey Replication. Arch Gen Psychiatry.

[2] James SL, Abate D, Abate KH, Abay SM, Abbafati C, Abbasi N (2018). Global, regional, and national incidence, prevalence, and years lived with disability for 354 diseases and injuries for 195 countries and territories, 1990–2017: a systematic analysis for the Global Burden of Disease Study 2017. Lancet.

[3] Dantzer R, O’Connor JC, Freund GG, Johnson RW, Kelley KW (2008). From inflammation to sickness and depression: when the immune system subjugates the brain. Nat Rev Neurosci..

[4] Miller AH (2009). Mechanisms of cytokine-induced behavioral changes: psychoneuroimmunology at the translational interface. Brain Behav Immun..

[5] Slavich GM, Irwin M (2014). From stress to inflammation and major depressive disorder: a social signal transduction theory of depression. Psychol Bull..

[6] Osimo EF, Pillinger T, Rodriguez IM, Khandaker GM, Pariante CM, Howes OD (2020). Inflammatory markers in depression: a meta-analysis of mean differences and variability in 5,166 patients and 5,083 controls. Brain Behav Immun..

[7] Eisenberger NI, Berkman ET, Inagaki TK, Rameson LT, Mashal NM, Irwin MR (2010). Inflammation-induced anhedonia: endotoxin reduces ventral striatum responses to reward. Biol Psychiatry.

[8] Dooley LN, Kuhlman KR, Robles TF, Eisenberger NI, Craske MG, Bower JE (2018). The role of inflammation in core features of depression: Insights from paradigms using exogenously-induced inflammation. Neurosci Biobehav Rev..

[9] DellaGioia N, Hannestad J (2010). A critical review of human endotoxin administration as an experimental paradigm of depression. Neurosci Biobehav Rev..

[10] Gimeno D, Kivimäki M, Brunner EJ, Elovainio M, De Vogli R, Steptoe A (2009). Associations of C-reactive protein and interleukin-6 with cognitive symptoms of depression: 12-year follow-up of the Whitehall II study. Psychol Med.

[11] Giollabhui N, Ng TH, Ellman LM, Alloy LB (2021). The longitudinal associations of inflammatory biomarkers and depression revisited: systematic review, meta-analysis, and meta-regression. Mol Psychiatry.

[12] Raison CL (2020). Microglial activation and response to anti-inflammatory treatment in major depressive disorder: another piece in the inflammation-mood disorders puzzle. Biol Psychiatry.

[13] Raison CL, Miller AH (2011). Is depression an inflammatory disorder?. Curr Psychiatry Rep..

[14] Musselman DL, Lawson DH, Gumnick JF, Manatunga AK, Penna S, Goodkin RS (2001). Paroxetine for the prevention of depression induced by high-dose interferon alfa. N Engl J Med..

[15] Irwin MR, Piber D (2018). Insomnia and inflammation: a two hit model of depression risk and prevention. World Psychiatry.

[16] Irwin MR, Cole S, Olmstead R, Breen EC, Cho JJ, Moieni M (2019). Moderators for depressed mood and systemic and transcriptional inflammatory responses: a randomized controlled trial of endotoxin. Neuropsychopharmacology.

[17] Moieni M, Irwin MR, Jevtic I, Olmstead R, Breen EC, Eisenberger NI (2015). Sex differences in depressive and socioemotional responses to an inflammatory challenge: implications for sex differences in depression. Neuropsychopharmacology.

[18] Cho JH-J, Irwin MR, Eisenberger NI, Lamkin DM, Cole SW (2019). Transcriptomic predictors of inflammation-induced depressed mood. Neuropsychopharmacology.

[19] Cho HJ, Eisenberger NI, Olmstead R, Breen EC, Irwin MR (2016). Preexisting mild sleep disturbance as a vulnerability factor for inflammation-induced depressed mood: a human experimental study. Transl Psychiatry.

[20] Felger JC, Alagbe O, Pace TW, Woolwine BJ, Hu F, Raison CL (2011). Early activation of p38 mitogen-activated protein kinase is associated with interferon-alpha-induced depression and fatigue. Brain Behav Immun..

[21] Stieglitz J, Trumble BC, Thompson ME, Blackwell AD, Kaplan H, Gurven M (2015). Depression as sickness behavior? A test of the host defense hypothesis in a high pathogen population. Brain Behav Immun..

[22] Bonaccorso S, Puzella A, Marino V, Pasquini M, Biondi M, Artini M (2001). Immunotherapy with interferon-alpha in patients affected by chronic hepatitis C induces an intercorrelated stimulation of the cytokine network and an increase in depressive and anxiety symptoms. Psychiatry Res.

[23] Krueger C, Hawkins K, Wong S, Enns MW, Minuk G, Rempel JD (2011). Persistent pro-inflammatory cytokines following the initiation of pegylated IFN therapy in hepatitis C infection is associated with treatment-induced depression. J Viral Hepat..

[24] Raison CL, Borisov AS, Majer M, Drake DF, Pagnoni G, Woolwine BJ (2009). Activation of central nervous system inflammatory pathways by interferon-alpha: relationship to monoamines and depression. Biol Psychiatry.

[25] Capuron L, Raison CL, Musselman DL, Lawson DH, Nemeroff CB, Miller AH (2003). Association of exaggerated HPA axis response to the initial injection of interferon-alpha with development of depression during interferon-alpha therapy. Am J Psychiatry.

[26] Felger JC, Haroon E, Woolwine BJ, Raison CL, Miller AH (2016). Interferon-alpha-induced inflammation is associated with reduced glucocorticoid negative feedback sensitivity and depression in patients with hepatitis C virus. Physiol Behav..

[27] Capuron L, Ravaud A, Miller AH, Dantzer R (2004). Baseline mood and psychosocial characteristics of patients developing depressive symptoms during interleukin-2 and/or interferon-alpha cancer therapy. Brain Behav Immun..

[28] Franzen PL, Buysse DJ, Rabinovitz M, Pollock BG, Lotrich FE (2010). Poor sleep quality predicts onset of either major depression or subsyndromal depression with irritability during interferon-alpha treatment. Psychiatry Res.

[29] Lotrich FE, Rabinovitz M, Gironda P, Pollock BG (2007). Depression following pegylated interferon-alpha: characteristics and vulnerability. J Psychosom Res..

[30] Kuhlman KR, Robles TF, Haydon MD, Dooley L, Boyle CC, Bower JE (2020). Early life stress sensitizes individuals to the psychological correlates of mild fluctuations in inflammation. Dev Psychobiol..

[31] Miller GE, Cole SW (2012). Clustering of depression and inflammation in adolescents previously exposed to childhood adversity. Biol Psychiatry.

[32] Manigault AW, Kuhlman KR, Irwin MR, Cole SW, Ganz PA, Crespi CM, Vulnerability to inflammation-related depressive symptoms: moderation by stress in women with breast cancer. Brain Behav Immun. 2021. 10.1016/j.bbi.2021.03.004. 10.1016/j.bbi.2021.03.004 PMC8058308 33705868

[33] Ganz PA, Petersen L, Castellon SA, Bower JE, Silverman DH, Cole SW (2014). Cognitive function after the initiation of adjuvant endocrine therapy in early-stage breast cancer: an observational cohort study. J Clin Oncol..

[34] Ganz PA, Kwan L, Castellon SA, Oppenheim A, Bower JE, Silverman DH (2013). Cognitive complaints after breast cancer treatments: examining the relationship with neuropsychological test performance. JNCI J Natl Cancer Inst.

[35] Van Dyk K, Crespi CM, Bower JE, Castellon SA, Petersen L, Ganz PA (2019). The cognitive effects of endocrine therapy in survivors of breast cancer: a prospective longitudinal study up to 6 years after treatment. Cancer.

[36] Ganz PA, Petersen L, Bower JE, Crespi CM (2016). Impact of adjuvant endocrine therapy on quality of life and symptoms: observational data over 12 months from the mind-body study. J Clin Oncol J Am Soc Clin Oncol..

[37] Wittchen H-U, Hoyer J, Friis R (2001). Generalized anxiety disorder—a risk factor for depression. Int J Methods Psychiatr Res..

[38] Kendler KS, Gatz M, Gardner CO, Pedersen NL (2006). Personality and major depression: a Swedish longitudinal, population-based twin study. Arch Gen Psychiatry.

[39] Baglioni C, Battagliese G, Feige B, Spiegelhalder K, Nissen C, Voderholzer U (2011). Insomnia as a predictor of depression: a meta-analytic evaluation of longitudinal epidemiological studies. J Affect Disord..

[40] Mazure CM (1998). Life stressors as risk factors in depression. Clin Psychol Sci Pract..

[41] Kendler KS, Karkowski LM, Prescott CA (1999). Causal relationship between stressful life events and the onset of major depression. Am J Psychiatry.

[42] Linden W, Vodermaier A, MacKenzie R, Greig D (2012). Anxiety and depression after cancer diagnosis: prevalence rates by cancer type, gender, and age. J Affect Disord..

[43] DiMatteo MR, Giordani PJ, Lepper HS, Croghan TW (2002). Patient adherence and medical treatment outcomes: a meta-analysis. Med Care.

[44] Goldstein D, Bennett BK, Webber K, Boyle F, De Souza PL, Wilcken NR (2012). Cancer-related fatigue in women with breast cancer: outcomes of a 5-year prospective cohort study. J Clin Oncol.

[45] Pinquart M, Duberstein PR (2010). Depression and cancer mortality: a meta-analysis. Psychol Med..

[46] Bower JE, Ganz PA, Irwin MR, Kwan L, Breen EC, Cole SW (2011). Inflammation and behavioral symptoms after breast cancer treatment: do fatigue, depression, and sleep disturbance share a common underlying mechanism?. J Clin Oncol..

[47] Miller AH, Ancoli-Israel S, Bower JE, Capuron L, Irwin MR (2008). Neuroendocrine-immune mechanisms of behavioral comorbidities in patients with cancer. J Clin Oncol..

[48] Stanton AL, Rowland JH, Ganz PA (2015). Life after diagnosis and treatment of cancer in adulthood: contributions from psychosocial oncology research. Am Psychol..

[49] Miller GE, White SF, Chen E, Nusslock R (2021). Association of inflammatory activity with larger neural responses to threat and reward among children living in poverty. Am J Psychiatry.

[50] Beck AT, Steer RA, Brown GK. Manual for the Beck Depression Inventory-II. *The Psychological Corporation.* 1996.

[51] Spielberger CD, Gorsuch RL, Lushene RE. Manual for the state-trait anxiety inventory. *Consulting Psychologist.* 1970.

[52] Cohen S, Kamarck T, Mermelstein R (1983). A global measure of perceived stress. J Health Soc Behav..

[53] Watson D, Clark LA, Tellegen A (1988). Development and validation of brief measures of positive and negative affect: the PANAS scales. J Pers Soc Psychol..

[54] Buysse DJ, Reynolds CF, Monk TH, Hoch CC, Yeager AL, Kupfer DJ (1991). Quantification of subjective sleep quality in healthy elderly men and women using the Pittsburgh Sleep Quality Index (PSQI). Sleep.

[55] Taylor SE, Lerner JS, Sage RM, Lehman BJ, Seeman TE (2004). Early environment, emotions, responses to stress, and health. J Pers..

[56] O’Connor MF, Bower JE, Cho HJ, Creswell JD, Dimitrov S, Hamby ME (2009). To assess, to control, to exclude: effects of biobehavioral factors on circulating inflammatory markers. Brain Behav Immun..

[57] Koller M (2016). robustlmm: An R package for robust estimation of linear mixed-effects models. J Stat Softw..

[58] R Core Team. R: A language and environment for statistical computing. R Foundation for Statistical Computing; Vienna, Austria. 2019.

[59] Tofighi D, MacKinnon DP (2011). RMediation: An R package for mediation analysis confidence intervals. Behav Res Methods.

[60] Lorah J (2018). Effect size measures for multilevel models: definition, interpretation, and TIMSS example. Large-Scale Assess Educ.

[61] Holm S (1979). A simple sequentially rejective multiple test procedure. Scand J Stat.

[62] Lüdecke D (2021). sjPlot: Data Visualization for Statistics in Social Science. R package version 2.8.10, https://CRAN.R-project.org/package=sjPlot.

[63] Knight RG, Waal‐Manning HJ, Spears GF (1983). Some norms and reliability data for the State-Trait Anxiety Inventory and the Zung Self-Rating Depression scale.. Br J Clin Psychol.

[64] Julian LJ. Measures of anxiety. Arthritis Care Res. 2011; 63(0 11). 10.1002/acr.20561 PMC3879951 22588767

[65] Smith KJ, Norris S, O’Farrelly C, O’Mara SM (2011). Risk factors for the development of depression in patients with hepatitis C taking interferon-α. Neuropsychiatr Dis Treat..

[66] Miller GE, Chen E, Parker KJ (2011). Psychological stress in childhood and susceptibility to the chronic diseases of aging: moving towards a model of behavioral and biological mechanisms. Psychol Bull..

[67] Dudek KA, Dion-Albert L, Lebel M, LeClair K, Labrecque S, Tuck E (2020). Molecular adaptations of the blood-brain barrier promote stress resilience vs. depression. Proc Natl Acad Sci USA..

[68] Wohleb ES, Powell ND, Godbout JP, Sheridan JF (2013). Stress-induced recruitment of bone marrow-derived monocytes to the brain promotes anxiety-like behavior. J Neurosci..

[69] Frank MG, Fonken LK, Watkins LR, Maier SF. Acute stress induces chronic neuroinflammatory, microglial, and behavioral priming: a role for potentiated NLRP3 inflammasome activation. Brain Behav Immun. 2020. 10.1016/j.bbi.2020.05.063. 10.1016/j.bbi.2020.05.063 PMC7572608 32485293

[70] Gomez-Gonzalez B, Hurtado-Alvarado G, Esqueda-Leon E, Santana-Miranda R, Rojas-Zamorano JA, Velazquez-Moctezuma J (2013). REM sleep loss and recovery regulates blood-brain barrier function. Curr Neurovasc Res..

[71] Wohleb ES, Patterson JM, Sharma V, Quan N, Godbout JP, Sheridan JF (2014). Knockdown of interleukin-1 receptor type-1 on endothelial cells attenuated stress-induced neuroinflammation and prevented anxiety-like behavior. J Neurosci J Soc Neurosci..

[72] Wohleb ES, Hanke ML, Corona AW, Powell ND, La'Tonia MS, Bailey MT (2011). β-Adrenergic receptor antagonism prevents anxiety-like behavior and microglial reactivity induced by repeated social defeat. J Neurosci J Soc Neurosci..

[73] Wichers M, Myin-Germeys I, Jacobs N, Peeters F, Kenis G, Derom C (2007). Genetic risk of depression and stress-induced negative affect in daily life. Br J Psychiatry.

[74] Ganz PA, Bower JE, Kwan L, Castellon SA, Silverman DHS, Geist C (2013). Does tumor necrosis factor-alpha (TNF-α) play a role in post-chemotherapy cerebral dysfunction?. Brain Behav Immun..

[75] Bower JE, Ganz PA, Aziz N, Fahey JL (2002). Fatigue and proinflammatory cytokine activity in breast cancer survivors. Psychosom Med..

[76] Howard-Anderson J, Ganz PA, Bower JE, Stanton AL (2012). Quality of life, fertility concerns, and behavioral health outcomes in younger breast cancer survivors: a systematic review. JNCI J Natl Cancer Inst..

[77] Grivennikov SI, Greten FR, Karin M (2010). Immunity, inflammation, and cancer. Cell.

[78] Bower JE, Lamkin DM (2013). Inflammation and cancer-related fatigue: mechanisms, contributing factors, and treatment implications. Brain Behav Immun..

[79] Jehn CF, Flath B, Strux A, Krebs M, Possinger K, Pezzutto A (2012). Influence of age, performance status, cancer activity, and IL-6 on anxiety and depression in patients with metastatic breast cancer. Breast Cancer Res Treat.

[80] Torres MA, Pace TW, Liu T, Felger JC, Mister D, Doho GH (2013). Predictors of depression in breast cancer patients treated with radiation: role of prior chemotherapy and nuclear factor kappa B. Cancer.

