# Supplementary material for: Moderators of inflammation-related depression: a prospective study of breast cancer survivors
Source: Transl Psychiatry. 2021 Dec 6;11:615. doi: 10.1038/s41398-021-01744-6 (PMC8648787; doi:10.1038/s41398-021-01744-6)
Supplement: Supplementary file 1 — Supplemental Materials [file 41398_2021_1744_MOESM1_ESM.docx]

**Supplemental Materials**

**Moderating Effects of Risk Factors Beyond T1**

Primary analyses focused on T1 risk factor levels. Secondary analyses were conducted to determine whether primary results were specific to T1 risk factors, or whether similar results may be obtained when examining the within- and between-subject variability in these risk factors across time. In other words, is exhibiting higher anxiety, stress, negative affect, or sleep disturbance than one’s average across time predictive of the inflammation-depressive symptoms link, or is exhibiting greater average anxiety, stress, negative affect, or sleep disturbance than other individuals in the sample predictive of the inflammation-depressive symptoms link. These analyses revealed a significant interaction of person-centered negative affect by person-centered inflammation index scores predicting depressive symptoms (b = .12, SE = .04, β = .06, t(451) = 2.74, *p* = .006), such that the link between person-centered inflammation and depressive symptoms was stronger on sampling occasions when negative affect was higher than typical. By contrasts, person-centered anxiety (b = .04, SE = .04, β = .03, t(426) = 1.12, *p* = .26), perceived stress (b = .03, SE = .04, β = .02, t(466) = .61, *p* = .54), and sleep disturbance (b = .01, SE = .08, β = .002, t(431) = .09, *p* = .93) did not interact with person-centered inflammation to predict depressive symptoms. Finally, all interaction tests examining between-person variability in risk factors were non-significant (anxiety: b = .02, SE = .02, β = .02, t(386) = .90, *p* = .37; perceived stress: b = .03, SE = .02, β = .03, t(385) = 1.34, *p* = .18; negative affect: b = .001, SE = .03, β = .001, t(387) = .05, *p* = .96; sleep disturbance: b = .07, SE = .05, β = .03, t(382) = 1.49, *p* = .14).

**Test of Between-Subject Associations**

Primary analyses focused on within-person associations between inflammation and depression, but between-subject associations may also be of interest. Controlling for covariates, tests of between-person associations revealed that total inflammation across time was not significantly associated with total depressive symptoms across time (*b* = .32, *SE* = .28, β = .09, *t*(183) = 1.16, *p* = .25), suggesting that exhibiting greater inflammation across time (than other participants in the study) was not significantly associated with greater depression across time. This association was not moderated by any risk factor examined (all *p*s > .05). Finally, results of primary analyses (i.e., interactions of risk factors with the within-subject association between inflammation and depression) remained unchanged when including the between-person effect in the model or controlling for interactions of risk factors with the between-subject association between inflammation and depression.

**Relative Contribution of Risk Factors**

Some risk factors may be particularly strong indicators of vulnerability to inflammation-associated depression. To evaluate this possibility, 2-way interactions of inflammation with T1 anxiety, T1 perceived stress, T1 negative affect, T1 sleep disturbance, and T3 childhood adversity were all entered into a single model, such that the degree of shared variance explained could be examined. When controlling for other 2-way interactions in the model, none of the risk factors examined significantly interacted with inflammation to predict depressive symptoms (anxiety interaction: b = -.016, SE = .03, β = -.02, t(363) = .52, *p* = .60; perceived stress interaction: b = .04, SE = .04, β = .05, t(363) = 1.13, *p* = .26; negative affect interaction: b = .02, SE = .04, β = .02, t(363) = .47, *p* = .64; sleep disturbance interaction: b = .09, SE = .05, β = .05, t(363) = 1.94, *p* = .052; childhood adversity interaction: b = -.005, SE = .02, β = -.01, t(363) = .32, *p* = .75), suggesting that no single factor predicted vulnerability for inflammation-associated depression above and beyond all others.

**Relative Contribution of Inflammatory Markers**

As shown on Table S3 and Figure S1, examining each inflammatory marker (i.e., CRP, IL-6, and sTNF-RII) individually revealed a consistent pattern of results where greater levels of state anxiety, perceived stress, negative affect, and sleep disturbance rendered the association between each inflammatory marker and depressive symptoms more positive (i.e., all interaction βs > 0). Notably, tests of interactions and simple slopes reliably reached statistical significance only for sTNF-RII. The association between person-centered sTNF-RII and depressive symptoms was significantly moderated by state anxiety (b = .31, SE = .14, β = .05, t(395) = 2.20, *p* = .028), perceived stress (b = .58, SE = .18, β = .07, t(395) = 3.31, *p* = .001), negative affect (b = .53, SE = .20, β = .05, t(397) = 2.60, *p* = .010), and sleep disturbance (b = 1.03, SE = .34, β = .06, t(387) = 3.00, *p* = .003), such that sTNF-RII was positively associated with depressive symptoms among women who reported average (mean) or high (+1 SD) levels of anxiety (b = 2.70, SE = 1.21, β = .05, t(394) = 2.23, *p* = .026, and b = 5.29, SE = 1.66, β = .09, t(397) = 3.20, *p* = .002, respectively), perceived stress (b = 3.06, SE = 1.18, β = .05, t(395) = 2.60, *p* = .010, and b = 6.98, SE = 1.70, β = .12, t(397) = 4.11, *p* < .001, respectively), negative affect (b = 2.46, SE = 1.20, β = .04, t(395) = 2.06, *p* = .040, and b = 5.53, SE = 1.62, β = .10, t(399) = 3.41, *p* < .001, respectively) and sleep disturbance (b = 2.74, SE = 1.19, β = .05, t(387) = 2.30, *p* = .022, and b = 6.33, SE = 1.72, β = .11, t(388) = 3.68, *p* < .001, respectively). By contrast, the association between person-centered IL-6 and depressive symptoms was significantly moderated by sleep disturbance (b = .31, SE = .14, β = .05, t(381) = 2.24, *p* = .026; non-significant under Holm’s adjustment), but only marginally moderated by state anxiety (b = .11, SE = .07, β = .04, t(388) = 1.68, *p* = .093), perceived stress (b = .14, SE = .08, β = .04, t(389) = 1.84, *p* = .066), and negative affect (b = .16, SE = .09, β = .04, t(390) = 1.73, *p* = .084), such that person-centered IL-6 was positively associated with depressive symptoms among women who reported high (+1 SD) levels of anxiety (b = 1.77, SE = .76, β = .08, t(391) = 2.32, *p* = .021), perceived stress (b = 1.79, SE = .71, β = .08, t(391) = 2.52, *p* = .012), negative affect (b = 1.78, SE = .74, β = .08, t(393) = 2.40, *p* = .017) and sleep disturbance (b = 1.71, SE = .65, β = .08, t(385) = 2.64, *p* = .009). Finally, the association between person-centered CRP and depressive symptoms was marginally moderated by state anxiety (b = .06, SE = .03, β = .04, t(388) = 1.68, *p* = .093), and perceived stress (b = .08, SE = .04, β = .04, t(389) = 1.82, *p* = .069), but all tests of simple slopes were non-significant (all *p*s > .05).

**Interactions of Age by Inflammation by Risk Factors**

We investigated whether results of primary analyses (i.e., interactive effects of inflammation and risk factors) were comparable across breast cancer survivors of varied ages. These analyses revealed significant 3-way interactions of age by state anxiety by inflammation (b = -.005, SE = .002, β = -.05, *t*(379) = 2.40, *p* = .017), age by perceived stress by inflammation (b = -.005, SE = .002, β = -.05, *t*(379) = 2.13, *p* = .033), age by sleep disturbance by inflammation (b = -.013, SE = .005, β = -.06, *t*(374) = 2.82, *p* = .005), and a marginally significant interaction of age by negative affect by inflammation (b = -.005, SE = .003, β = -.04, *t*(381) = 1.77, *p* = .078). By contrast, childhood adversity did not interact with age and inflammation to predict depressive symptoms (b = -.0008, SE = .002, β = -.01, *t*(371) = .41, *p* = .68). Follow up tests contrasted results across levels of the age variable (-1SD: 43.5 years; mean: 51.6 years; +1SD: 59.7 years). As shown on Figure S2, states anxiety, perceived stress, and sleep disturbance significantly interacted with inflammation to predict depressive symptoms among younger (-1SD) women (states anxiety: b = .08, SE = .02, β = .11, t(380) = 3.49, *p* < .001; perceived stress : b = .11, SE = .03, β = .12, t(380) = 3.83, *p* < .001; sleep disturbance: b = .23, SE = .06, β = .13, t(377) = 3.95, *p* < .001) and women of average (mean) age (states anxiety: b = .04, SE = .02, β = .06, t(380) = 2.49, *p* = .013; perceived stress : b = .07, SE = .02, β = .08, t(380) = 3.22, *p* = .001; sleep disturbance: b = .12, SE = .04, β = .07, t(375) = 2.97, *p* = .003). By contrast, states anxiety, perceived stress, and sleep disturbance did not interact with inflammation to predict depressive symptoms among older (+1SD) women (states anxiety: b = .005, SE = .02, β = .007, t(379) = .23, *p* = .82; perceived stress : b = .03, SE = .03, β = .03, t(379) = .97, *p* = .33; sleep disturbance: b = .01, SE = .05, β = .006, t(372) = .19, *p* = .85). Similarly, negative affect interacted with inflammation to predict depressive symptoms among younger (-1SD) women (b = .08, SE = .03, β = .07, t(384) = 2.65, *p* = .008), but not among women of average (mean) age or older (+1SD) women (b = .04, SE = .03, β = .03, t(384) = 1.41, *p* = .16, and b = -.003, SE = .04, β = -.003, t(382) = .08, *p* = .93, respectively). Tests of simple slopes revealed that inflammation was positively associated with depressive symptoms among younger (-1SD) women and women of average (mean) age who reported high (+1SD) state anxiety (b = .81, SE = .22, β = .13, t(385) = 3.66, *p* < .001, and b = .45, SE = .20, β = .07, t(383) = 2.22, *p* = .027, respectively), perceived stress (b = .98, SE = .23, β = .16, t(385) = 4.28, *p* < .001, and b = .62, SE = .21, β = .10, t(384) = 3.00, *p* = .003, respectively) and sleep disturbance (b = 1.08, SE = .25, β = .18, t(383) = 4.33, *p* < .001, and b = .51, SE = .19, β = .08, t(380) = 2.64, *p* = .009, respectively). Similarly, inflammation was positively associated with depressive symptoms among younger (-1SD) women who reported high (+1SD) negative affect (b = .66, SE = .21, β = .11, t(389) = 3.09, *p* = .002). All other tests of simple slopes were non-significant (*p*s > .05).

**Accounting for overlap between BDI-II items and measures of anxiety/sleep disturbance**

The questionnaire used to assess depressive symptoms includes 1 item pertaining to anxiety (i.e., “agitation”) and 1 item pertaining to sleep (i.e., “changes in sleeping pattern”). Primary analyses were thereby repeated using a depressive symptom sum score that does not include these two items. This change did not alter primary conclusion of the study; the association between person-centered inflammation and depressive symptoms (excluding the agitation and sleep items) at any given time was moderated by post-treatment (T1) levels of state anxiety (b = .05, SE = .02, β = .08, *t*(373) = 3.36, *p* < .001), perceived stress (b = .08, SE = .02, β = .09, *t*(372) = 4.02, *p* < .001), negative affect (b = .07, SE = .02, β = .07, *t*(376) = 3.07, *p* = .002) and sleep disturbance (b = .10, SE = .03, β = .06, *t*(365) = 2.98, *p* = .003).

**Additional Tests of Demographic and Treatment Variables**

Demographic and disease/treatment-related variables (i.e., age, education, race/ethnicity, marital status, income, cancer stage, surgery type, receipt of chemotherapy, receipt of radiation therapy, and receipt of endocrine therapy) were examined among individual who scored low/high on depressive symptoms (Table S4) and the inflammation index (Table S5) across the assessment period (T1-TF), as well as those who scored low/high on T1 anxiety (Table S6), T1 perceived stress (Table S7), T1 negative affect (Table S8), and T1 sleep disturbance (Table S9). Published cutoff scores were used for depression (> 13)^1^, anxiety (> 40)^2^, perceived stress (> 13)^3^, and sleep disturbance (> 5)^4^, whereas median splits were used for the inflammation index and negative affect (given that cutoff scores were not available for these measures). These analyses revealed that survivors who scored above the median on the inflammation index were older (*t*(158.4) = 2.9, *p* = .005), more likely to present with stage 2 or 3 breast cancer relative to stage 0 or 1 (*X^2^*(1, 161) = 5.3, *p* = .02), and were more likely to report “less than a college degree” relative to “College degree” (*X^2^*(1, 82) = 7.1, *p* = .008). Survivors who scored above the median on T1 perceived stress were younger (*t*(157) = 2.1, *p* = .03). Finally, survivors who scored above the median on T1 sleep disturbance were more likely to exhibit stage 2 or 3 breast cancer relative to stage 0 or 1 (*X^2^*(1, 157) = 4.7, *p* = .03), and more likely to report receipt of chemotherapy relative to no chemotherapy (*X^2^*(1, 157) = 10.1, *p* < .01).

**Supplemental References**

1 Beck AT, Steer RA, Brown GK. *Manual for the Beck Depression Inventory-II*. San Antonio, TX, 1996.

2 Julian LJ. Measures of Anxiety. *Arthritis Care Res* 2011; **63**. doi:10.1002/acr.20561.

3 Cohen S, Kamarck T, Mermelstein R. A Global Measure of Perceived Stress. *J Health Soc Behav* 1983; **24**: 385–396.

4 Buysse DJ, Reynolds CF III, Monk TH, Hoch Carolyn C, Yeager AL, Kupfer DJ. Quantification of Subjective Sleep Quality in Healthy Elderly Men and Women Using the Pittsburgh Sleep Quality Index (PSQI). *Sleep* 1991; **14**: 331–338.

**Supplemental Tables and Figures**

| **Table S1.**  Descriptive statistics of primary study variables by study time point. | | | | | | | | |
| --- | --- | --- | --- | --- | --- | --- | --- | --- |
|  | Post-treatment (T1) | | 6 months  (T2) | | 12 months  (T3) | | 3-6 years  (TF) | |
|  | Mean | (SD) | Mean | (SD) | Mean | (SD) | Mean | (SD) |
| Depressive Symptoms | 8.69 | (6.92) | 8.33 | (6.91) | 8.56 | (7.00) | 8.28 | (7.48) |
| Inflammation Index Scores | 0.49 | (2.21) | -0.27 | (2.05) | -0.37 | (2.18) | 0.02 | (2.19) |
| State Anxiety | 35.00 | (8.47) | 34.08 | (8.07) | 35.97 | (9.59) | 35.43 | (8.81) |
| Perceived Stress | 13.96 | (6.75) | 13.79 | (6.80) | 14.14 | (6.86) | 14.05 | (6.93) |
| Negative Affect | 16.28 | (5.86) | 16.08 | (5.90) | 16.37 | (6.20) | 15.48 | (5.91) |
| Sleep Disturbance | 7.54 | (3.49) | 6.50 | (3.40) | 7.26 | (3.64) | 6.75 | (3.77) |
| Childhood Adversity |  |  |  |  | 27.83 | (10.44) |  |  |
|  | | | | | | | | |

| **Table S2.**  Standardized coefficients for re-centered multilevel models predicting depressive symptoms as a function of the composite inflammatory index and key risk factors (anxiety, stress, negative affect and sleep disturbance). | | | | | | |
| --- | --- | --- | --- | --- | --- | --- |
|  | State Anxiety (SAI) | | | | | |
| SAI Centering | -1SD | | mean | | +1SD | |
|  | β | (SE) | β | (SE) | β | (SE) |
| Intercept | -.45* | (.13) | .13 | (.12) | .70* | (.13) |
| Inflammation | -.03 | (.03) | .03 | (.02) | .09* | (.03) |
| SAI | .58* | (.05) | .58* | (.05) | .58* | (.05) |
| Inflammation x SAI | .06* | (.02) | .06* | (.02) | .06* | (.02) |
|  | Perceived Stress (PSS) | | | | | |
| PSS Centering | -1SD | | mean | | +1SD | |
|  | β | (SE) | β | (SE) | β | (SE) |
| Intercept | -.41* | (.14) | .10 | (.13) | .62* | (.13) |
| Inflammation | -.04 | (.03) | .04^†^ | (.02) | .11* | (.03) |
| PSS | .52* | (.05) | .52* | (.05) | .52* | (.05) |
| Inflammation x PSS | .08* | (.02) | .08* | (.02) | .08* | (.02) |
|  | Negative Affect (NA) | | | | | |
| NA Centering | -1SD | | mean | | +1SD | |
|  | β | (SE) | β | (SE) | β | (SE) |
| Intercept | .40* | (.14) | .16 | (.13) | .73* | (.14) |
| Inflammation | -.02 | (.03) | .03 | (.02) | .09* | (.03) |
| NA | .57* | (.05) | .57* | (.05) | .57* | (.05) |
| Inflammation x NA | .06* | (.02) | .06* | (.02) | .06* | (.02) |
|  | Sleep Disturbance (PSQI) | | | | | |
| PSQI Centering | -1SD | | mean | | +1SD | |
|  | β | (SE) | β | (SE) | β | (SE) |
| Intercept | -.12 | (.16) | .18 | (.14) | .48* | (.16) |
| Inflammation | -.04 | (.03) | .03 | (.02) | .09* | (.03) |
| PSQI | .30* | (.06) | .30* | (.06) | .30* | (.06) |
| Inflammation x PSQI | .06* | (.02) | .06* | (.02) | .06* | (.02) |
| Notes: All models shown controlled for age, BMI, race, receipt of chemotherapy, radiation therapy, and endocrine therapy, cancer stage, and surgery type. ^†^p < .10; *p < .05; Inflammation = scores on the composite inflammatory index; SAI = state anxiety; PSS = perceived stress; NA = negative affect; PSQI = sleep disturbance. Risk factors were modeled as continuous measures in all models. | | | | | | |

| **Table S3.**  Standardized coefficients for re-centered multilevel models predicting depressive symptoms as a function of inflammation markers (CRP, IL-6, and sTNF-RII) and key risk factors (anxiety, stress, negative affect and sleep disturbance). | | | | | | | | | | | | | | | | | | |
| --- | --- | --- | --- | --- | --- | --- | --- | --- | --- | --- | --- | --- | --- | --- | --- | --- | --- | --- |
|  | State Anxiety (SAI) | | | | | | | | | | | | | | | | | |
| Inflammatory Marker | CRP | | | | | | IL-6 | | | | | | sTNF-RII | | | | | |
| SAI Centering | -1SD | | mean | | +1SD | | -1SD | | mean | | +1SD | | -1SD | | mean | | +1SD | |
|  | β | (SE) | β | (SE) | β | (SE) | β | (SE) | β | (SE) | β | (SE) | β | (SE) | β | (SE) | β | (SE) |
| Intercept | -.43* | (.13) | .14 | (.12) | .71* | (.13) | -.44* | (.13) | .13 | (.12) | .70* | (.13) | -.45* | (.13) | .13* | (.12) | .72* | (.13) |
| Inflammatory Marker | -.05 | (.03) | -.01 | (.02) | .03 | (.03) | -.00 | (.03) | .04^†^ | (.02) | .08* | (.76) | .00 | (.03) | .05* | (.02) | .09* | (.03) |
| SAI | .57* | (.05) | .57* | (.05) | .57* | (.05) | .57* | (.05) | .57* | (.05) | .57* | (.05) | .58* | (.05) | .58* | (.05) | .58* | (.05) |
| Inflammatory Marker x SAI | .04^†^ | (.02) | .04^†^ | (.02) | .04^†^ | (.02) | .04^†^ | (.03) | .04^†^ | (.03) | .04^†^ | (.03) | .05* | (.02) | .05* | (.02) | .05* | (.02) |
|  | Perceived Stress (PSS) | | | | | | | | | | | | | | | | | |
| Inflammatory Marker | CRP | | | | | | IL-6 | | | | | | sTNF-RII | | | | | |
| PSS Centering | -1SD | | mean | | +1SD | | -1SD | | mean | | +1SD | | -1SD | | mean | | +1SD | |
|  | β | (SE) | β | (SE) | β | (SE) | β | (SE) | β | (SE) | β | (SE) | β | (SE) | β | (SE) | β | (SE) |
| Intercept | -.40* | (.14) | .12 | (.13) | .63* | (.13) | -.40* | (.14) | .11 | (.13) | .62* | (.13) | -.41* | (.14) | .11 | (.13) | .64* | (.13) |
| Inflammatory Marker | -.05 | (.03) | -.00 | (.02) | .04 | (.03) | -.00 | (.03) | .04^†^ | (.02) | .08* | (.03) | -.02 | (.03) | .05* | (.02) | .12* | (.03) |
| PSS | .51* | (.05) | .51* | (.05) | .51* | (.05) | .50* | (.05) | .50* | (.05) | .50* | (.05) | .52* | (.05) | .52* | (.05) | .52* | (.05) |
| Inflammatory Marker x PSS | .04^†^ | (.02) | .04^†^ | (.02) | .04^†^ | (.02) | .04^†^ | (.02) | .04^†^ | (.02) | .04^†^ | (.02) | .07* | (.02) | .07* | (.02) | .07* | (.02) |
|  | Negative Affect (NA) | | | | | | | | | | | | | | | | | |
| Inflammatory Marker | CRP | | | | | | IL-6 | | | | | | sTNF-RII | | | | | |
| NA Centering | -1SD | | mean | | +1SD | | -1SD | | mean | | +1SD | | -1SD | | mean | | +1SD | |
|  | β | (SE) | β | (SE) | β | (SE) | β | (SE) | β | (SE) | β | (SE) | β | (SE) | β | (SE) | β | (SE) |
| Intercept | .39* | (.14) | .18 | (.13) | .74* | (.14) | .39* | (.14) | .17 | (13) | .73* | (.14) | -.41* | (.14) | .17 | (.13) | .74* | (.14) |
| Inflammatory Marker | -.03 | (.03) | -.01 | (.02) | .02 | (.03) | -.00 | (.03) | .04^†^ | (.02) | .08* | (.03) | -.01 | (.03) | .04* | (.02) | .10* | (.03) |
| NA | .56* | (.05) | .56* | (.05) | .56* | (.05) | .56* | (.05) | .56* | (.05) | .56* | (.05) | .57* | (.05) | .57* | (.05) | .57* | (.05) |
| Inflammatory Marker x NA | .03 | (.02) | .03 | (.02) | .03 | (.02) | .04^†^ | (.02) | .04^†^ | (.02) | .04^†^ | (.02) | .05* | (.02) | .05* | (.02) | .05* | (.02) |
|  | Sleep Disturbance (PSQI) | | | | | | | | | | | | | | | | | |
| Inflammatory Marker | CRP | | | | | | IL-6 | | | | | | sTNF-RII | | | | | |
| PSQI Centering | -1SD | | mean | | +1SD | | -1SD | | mean | | +1SD | | -1SD | | mean | | +1SD | |
|  | β | (SE) | β | (SE) | β | (SE) | β | (SE) | β | (SE) | β | (SE) | β | (SE) | β | (SE) | β | (SE) |
| Intercept | -.11 | (.16) | .19 | (.15) | .49* | (.16) | -.12 | (.16) | .18 | (.14) | .48* | (.15) | -.13 | (.16) | .18 | (.14) | .49* | (.16) |
| Inflammatory Marker | -.03 | (.03) | -.01 | (.02) | .02 | (.03) | -.02 | (.03) | .03 | (.02) | .08* | (.03) | -.02 | (.03) | .05* | (.02) | .11* | (.03) |
| PSQI | .30* | (.06) | .30* | (.06) | .30* | (.06) | .30* | (.06) | .30* | (.06) | .30* | (.06) | .31* | (.06) | .31* | (.06) | .31* | (.06) |
| Inflammatory Marker x PSQI | .03 | (.02) | .03 | (.02) | .03 | (.02) | .05* | (.02) | .05* | (.02) | .05* | (.02) | .06* | (.02) | .06* | (.02) | .06* | (.02) |
| Notes: All models shown controlled for age, BMI, race, receipt of chemotherapy, radiation therapy, and endocrine therapy, cancer stage, and surgery type. ^†^p < .10; *p < .05; CRP = c-reactive protein; IL-6 = interleukin 6; sTNF-RII = soluble tumor necrosis factor receptor 2; SAI = state anxiety; PSS = perceived stress; NA = negative affect; PSQI = sleep disturbance. Risk factors were modeled as continuous measures in all models. | | | | | | | | | | | | | | | | | | |

| **Table S4.**  Demographics and Clinical Characteristics of the Sample Compared on the Basis of Average Depressive Symptoms Across Time (T1-TF). | | | | | | | | | |
| --- | --- | --- | --- | --- | --- | --- | --- | --- | --- |
|  | Minimal Depressive Symptoms  (≤ 13) | | | | Above Minimal Depressive Symptoms (> 13) | | | |  |
| Variable | N | % | Mean | (SD) | N | % | Mean | (SD) | Comparison Statistic |
| Age (years) | 128 |  | 51.78 | (8.19) | 33 |  | 50.99 | (7.82) | *t*(51.6) = .51 |
| Education |  |  |  |  |  |  |  |  | *X^2^*(2, 161) = .52 |
| Less than College | 24 | 18.75% |  |  | 8 | 24.24% |  |  |  |
| College Degree | 40 | 31.25% |  |  | 10 | 30.3% |  |  |  |
| Post-Graduate Degree | 64 | 50% |  |  | 15 | 45.45% |  |  |  |
| Race/Ethnicity |  |  |  |  |  |  |  |  | *X^2^*(4, 161) = 5.4 |
| White/Caucasian | 102 | 79.69% |  |  | 24 | 72.73% |  |  |  |
| Hispanic/Latino | 13 | 10.16% |  |  | 4 | 12.12% |  |  |  |
| Black/African American | 4 | 3.13% |  |  | 1 | 3.03% |  |  |  |
| Asian | 7 | 5.47% |  |  | 1 | 3.03% |  |  |  |
| Other | 2 | 1.56% |  |  | 3 | 9.09% |  |  |  |
| Married |  |  |  |  |  |  |  |  | *X^2^*(1, 160) = .03 |
| Married or living as married | 84 | 66.14% |  |  | 23 | 69.7% |  |  |  |
| Divorced, separated, widowed, or never married | 43 | 33.86% |  |  | 10 | 30.3% |  |  |  |
| Income |  |  |  |  |  |  |  |  | *X^2^*(1, 157) = .21 |
| < $100,000 | 43 | 34.4% |  |  | 9 | 28.13% |  |  |  |
| > $100,000 | 82 | 65.6% |  |  | 23 | 71.88% |  |  |  |
| Breast Cancer Stage |  |  |  |  |  |  |  |  | *X^2^*(1, 161) = .09 |
| 0 or 1 | 76 | 59.38% |  |  | 18 | 54.55% |  |  |  |
| 2 or 3 | 52 | 40.63% |  |  | 15 | 45.45% |  |  |  |
| Surgery Type |  |  |  |  |  |  |  |  | *X^2^*(1, 161) = .46 |
| Lumpectomy | 88 | 68.75% |  |  | 20 | 60.61% |  |  |  |
| Mastectomy | 40 | 31.25% |  |  | 13 | 39.39% |  |  |  |
| Received chemotherapy |  |  |  |  |  |  |  |  | *X^2^*(1, 161) = .33 |
| Yes | 68 | 53.13% |  |  | 20 | 60.61% |  |  |  |
| No | 60 | 46.88% |  |  | 13 | 39.39% |  |  |  |
| Received radiation therapy |  |  |  |  |  |  |  |  | *X^2^*(1, 161) = 1.1 |
| Yes | 99 | 77.34% |  |  | 22 | 66.67% |  |  |  |
| No | 29 | 22.66% |  |  | 11 | 33.33% |  |  |  |
| Received endocrine therapy |  |  |  |  |  |  |  |  | *X^2^*(1, 161) = .24 |
| Yes | 93 | 72.66% |  |  | 26 | 78.79% |  |  |  |
| No | 35 | 27.34% |  |  | 7 | 21.21% |  |  |  |
| * denotes statistically significant comparison statistics (p < .05). | | | | | | | | | |

| **Table S5.**  Demographics and Clinical Characteristics of the Sample Compared on the Basis of Average Inflammation Index Scores Across Time (T1-TF). | | | | | | | | | |
| --- | --- | --- | --- | --- | --- | --- | --- | --- | --- |
|  | Low Inflammation Index  (≤ median^a^) | | | | High Inflammation Index  (> median^a^) | | | |  |
| Variable | N | % | Mean | (SD) | N | % | Mean | (SD) | Comparison Statistic |
| Age (years) | 81 |  | 49.83 | (7.72) | 80 |  | 53.42 | (8.12) | *t*(158.4) = 2.9* |
| Education |  |  |  |  |  |  |  |  | *X^2^*(2, 161) = 8.4* |
| Less than College | 10 | 12.35% |  |  | 22 | 27.5% |  |  |  |
| College Degree | 32 | 39.51% |  |  | 18 | 22.5% |  |  |  |
| Post-Graduate Degree | 39 | 48.15% |  |  | 40 | 50% |  |  |  |
| Race/Ethnicity |  |  |  |  |  |  |  |  | *X^2^*(4, 161) = 9.7* |
| White/Caucasian | 58 | 71.6% |  |  | 68 | 85% |  |  |  |
| Hispanic/Latino | 10 | 12.35% |  |  | 7 | 8.75% |  |  |  |
| Black/African American | 3 | 3.7% |  |  | 2 | 2.5% |  |  |  |
| Asian | 8 | 9.88% |  |  | 0 | 0% |  |  |  |
| Other | 2 | 2.47% |  |  | 3 | 3.75% |  |  |  |
| Married |  |  |  |  |  |  |  |  | *X^2^*(1, 160) < .01 |
| Married or living as married | 54 | 67.5% |  |  | 53 | 66.25% |  |  |  |
| Divorced, separated, widowed, or never married | 26 | 32.5% |  |  | 27 | 33.75% |  |  |  |
| Income |  |  |  |  |  |  |  |  | *X^2^*(1, 157) < .01 |
| < $100,000 | 26 | 33.33% |  |  | 26 | 32.91% |  |  |  |
| > $100,000 | 52 | 66.67% |  |  | 53 | 67.09% |  |  |  |
| Breast Cancer Stage |  |  |  |  |  |  |  |  | *X^2^*(1, 161) = 5.3* |
| 0 or 1 | 55 | 67.9% |  |  | 39 | 48.75% |  |  |  |
| 2 or 3 | 26 | 32.1% |  |  | 41 | 51.25% |  |  |  |
| Surgery Type |  |  |  |  |  |  |  |  | *X^2^*(1, 161) < .01 |
| Lumpectomy | 54 | 66.67% |  |  | 54 | 67.5% |  |  |  |
| Mastectomy | 27 | 33.33% |  |  | 26 | 32.5% |  |  |  |
| Received chemotherapy |  |  |  |  |  |  |  |  | *X^2^*(1, 161) = .06 |
| Yes | 43 | 53.09% |  |  | 45 | 56.25% |  |  |  |
| No | 38 | 46.91% |  |  | 35 | 43.75% |  |  |  |
| Received radiation therapy |  |  |  |  |  |  |  |  | *X^2^*(1, 161) = 1.5 |
| Yes | 57 | 70.37% |  |  | 64 | 80% |  |  |  |
| No | 24 | 29.63% |  |  | 16 | 20% |  |  |  |
| Received endocrine therapy |  |  |  |  |  |  |  |  | *X^2^*(1, 161) = 0.02 |
| Yes | 59 | 72.84% |  |  | 60 | 75% |  |  |  |
| No | 22 | 27.16% |  |  | 20 | 25% |  |  |  |
| * denotes statistically significant comparison statistics (p < .05). Median Inflammation Index = -.05. | | | | | | | | | |

| **Table S6.**  Demographics and Clinical Characteristics of the Sample Compared on the Basis of T1 State Anxiety. | | | | | | | | | |
| --- | --- | --- | --- | --- | --- | --- | --- | --- | --- |
|  | Low State Anxiety  (≤ 40) | | | | High State Anxiety  (> 40) | | | |  |
| Variable | N | % | Mean | (SD) | N | % | Mean | (SD) | Comparison Statistic |
| Age (years) | 118 |  | 52.23 | (7.82) | 42 |  | 50.02 | (8.78) | *t*(65.6) = 1.44 |
| Education |  |  |  |  |  |  |  |  | *X^2^*(2, 160) = .31 |
| Less than College | 24 | 20.34% |  |  | 8 | 19.05% |  |  |  |
| College Degree | 38 | 32.2% |  |  | 12 | 28.57% |  |  |  |
| Post-Graduate Degree | 56 | 47.46% |  |  | 22 | 52.38% |  |  |  |
| Race/Ethnicity |  |  |  |  |  |  |  |  | *X^2^*(4, 160) = 1.2 |
| White/Caucasian | 92 | 77.97% |  |  | 34 | 80.95% |  |  |  |
| Hispanic/Latino | 11 | 9.32% |  |  | 5 | 11.9% |  |  |  |
| Black/African American | 4 | 3.39% |  |  | 1 | 2.38% |  |  |  |
| Asian | 7 | 5.93% |  |  | 1 | 2.38% |  |  |  |
| Other | 4 | 3.39% |  |  | 1 | 2.38% |  |  |  |
| Married |  |  |  |  |  |  |  |  | *X^2^*(1, 160) = .03 |
| Married or living as married | 78 | 66.1% |  |  | 29 | 69.05% |  |  |  |
| Divorced, separated, widowed, or never married | 40 | 33.9% |  |  | 13 | 30.95% |  |  |  |
| Income |  |  |  |  |  |  |  |  | *X^2^*(1, 157) = .17 |
| < $100,000 | 40 | 34.48% |  |  | 12 | 29.27% |  |  |  |
| > $100,000 | 76 | 65.52% |  |  | 29 | 70.73% |  |  |  |
| Breast Cancer Stage |  |  |  |  |  |  |  |  | *X^2^*(1, 160) = 1.1 |
| 0 or 1 | 72 | 61.02% |  |  | 21 | 50% |  |  |  |
| 2 or 3 | 46 | 38.98% |  |  | 21 | 50% |  |  |  |
| Surgery Type |  |  |  |  |  |  |  |  | *X^2^*(1, 160) = 3.1 |
| Lumpectomy | 84 | 71.19% |  |  | 23 | 54.76% |  |  |  |
| Mastectomy | 34 | 28.81% |  |  | 19 | 45.24% |  |  |  |
| Received chemotherapy |  |  |  |  |  |  |  |  | *X^2^*(1, 160) = .92 |
| Yes | 61 | 51.69% |  |  | 26 | 61.9% |  |  |  |
| No | 57 | 48.31% |  |  | 16 | 38.1% |  |  |  |
| Received radiation therapy |  |  |  |  |  |  |  |  | *X^2^*(1, 160) = 1.5 |
| Yes | 92 | 77.97% |  |  | 28 | 66.67% |  |  |  |
| No | 26 | 22.03% |  |  | 14 | 33.33% |  |  |  |
| Received endocrine therapy |  |  |  |  |  |  |  |  | *X^2^*(1, 160) = 0.87 |
| Yes | 85 | 72.03% |  |  | 34 | 80.95% |  |  |  |
| No | 33 | 27.97% |  |  | 8 | 19.05% |  |  |  |
| * denotes statistically significant comparison statistics (p < .05). | | | | | | | | | |

| **Table S7.**  Demographics and Clinical Characteristics of the Sample Compared on the Basis of T1 Perceived Stress. | | | | | | | | | |
| --- | --- | --- | --- | --- | --- | --- | --- | --- | --- |
|  | Low Perceived Stress  (≤ 13) | | | | High Perceived Stress  (> 13) | | | |  |
| Variable | N | % | Mean | (SD) | N | % | Mean | (SD) | Comparison Statistic |
| Age (years) | 82 |  | 52.98 | (7.89) | 78 |  | 50.25 | (8.17) | *t*(156.9) = 2.15* |
| Education |  |  |  |  |  |  |  |  | *X^2^*(2, 160) = .44 |
| Less than College | 16 | 19.51% |  |  | 16 | 20.51% |  |  |  |
| College Degree | 24 | 29.27% |  |  | 26 | 33.33% |  |  |  |
| Post-Graduate Degree | 42 | 51.22% |  |  | 36 | 46.15% |  |  |  |
| Race/Ethnicity |  |  |  |  |  |  |  |  | *X^2^*(4, 160) = 2.1 |
| White/Caucasian | 66 | 80.49% |  |  | 60 | 76.92% |  |  |  |
| Hispanic/Latino | 6 | 7.32% |  |  | 10 | 12.82% |  |  |  |
| Black/African American | 3 | 3.66% |  |  | 2 | 2.56% |  |  |  |
| Asian | 5 | 6.1% |  |  | 3 | 3.85% |  |  |  |
| Other | 2 | 2.44% |  |  | 3 | 3.85% |  |  |  |
| Married |  |  |  |  |  |  |  |  | *X^2^*(1, 160) = 1.3 |
| Married or living as married | 51 | 62.2% |  |  | 56 | 71.79% |  |  |  |
| Divorced, separated, widowed, or never married | 31 | 37.8% |  |  | 22 | 28.21% |  |  |  |
| Income |  |  |  |  |  |  |  |  | *X^2^*(1, 157) = 1.6 |
| < $100,000 | 31 | 38.27% |  |  | 21 | 27.63% |  |  |  |
| > $100,000 | 50 | 61.73% |  |  | 55 | 72.37% |  |  |  |
| Breast Cancer Stage |  |  |  |  |  |  |  |  | *X^2^*(1, 160) = .83 |
| 0 or 1 | 51 | 62.2% |  |  | 42 | 53.85% |  |  |  |
| 2 or 3 | 31 | 37.8% |  |  | 36 | 46.15% |  |  |  |
| Surgery Type |  |  |  |  |  |  |  |  | *X^2^*(1, 160) = .05 |
| Lumpectomy | 56 | 68.29% |  |  | 51 | 65.38% |  |  |  |
| Mastectomy | 26 | 31.71% |  |  | 27 | 34.62% |  |  |  |
| Received chemotherapy |  |  |  |  |  |  |  |  | *X^2^*(1, 160) = 2.6 |
| Yes | 39 | 47.56% |  |  | 48 | 61.54% |  |  |  |
| No | 43 | 52.44% |  |  | 30 | 38.46% |  |  |  |
| Received radiation therapy |  |  |  |  |  |  |  |  | *X^2^*(1, 160) < .01 |
| Yes | 61 | 74.39% |  |  | 59 | 75.64% |  |  |  |
| No | 21 | 25.61% |  |  | 19 | 24.36% |  |  |  |
| Received endocrine therapy |  |  |  |  |  |  |  |  | *X^2^*(1, 160) = 0.03 |
| Yes | 62 | 75.61% |  |  | 57 | 73.08% |  |  |  |
| No | 20 | 24.39% |  |  | 21 | 26.92% |  |  |  |
| * denotes statistically significant comparison statistics (p < .05). | | | | | | | | | |

| **Table S8.**  Demographics and Clinical Characteristics of the Sample Compared on the Basis of T1 Negative Affect. | | | | | | | | | |
| --- | --- | --- | --- | --- | --- | --- | --- | --- | --- |
|  | Low Negative Affect  (≤ median^a^) | | | | High Negative Affect  (> median^a^) | | | |  |
| Variable | N | % | Mean | (SD) | N | % | Mean | (SD) | Comparison Statistic |
| Age (years) | 87 |  | 52.19 | (7.90) | 73 |  | 51.01 | (8.37) | *t*(149.8) = .91 |
| Education |  |  |  |  |  |  |  |  | *X^2^*(2, 160) = .68 |
| Less than College | 19 | 21.84% |  |  | 13 | 17.81% |  |  |  |
| College Degree | 28 | 32.18% |  |  | 22 | 30.14% |  |  |  |
| Post-Graduate Degree | 40 | 45.98% |  |  | 38 | 52.05% |  |  |  |
| Race/Ethnicity |  |  |  |  |  |  |  |  | *X^2^*(4, 160) = 1.1 |
| White/Caucasian | 69 | 79.31% |  |  | 57 | 78.08% |  |  |  |
| Hispanic/Latino | 7 | 8.05% |  |  | 9 | 12.33% |  |  |  |
| Black/African American | 3 | 3.45% |  |  | 2 | 2.74% |  |  |  |
| Asian | 5 | 5.75% |  |  | 3 | 4.11% |  |  |  |
| Other | 3 | 3.45% |  |  | 2 | 2.74% |  |  |  |
| Married |  |  |  |  |  |  |  |  | *X^2^*(1, 160) < .01 |
| Married or living as married | 58 | 66.67% |  |  | 49 | 67.12% |  |  |  |
| Divorced, separated, widowed, or never married | 29 | 33.33% |  |  | 24 | 32.88% |  |  |  |
| Income |  |  |  |  |  |  |  |  | *X^2^*(1, 157) < .01 |
| < $100,000 | 29 | 33.72% |  |  | 23 | 32.39% |  |  |  |
| > $100,000 | 57 | 66.28% |  |  | 48 | 67.61% |  |  |  |
| Breast Cancer Stage |  |  |  |  |  |  |  |  | *X^2^*(1, 160) = .39 |
| 0 or 1 | 53 | 60.92% |  |  | 40 | 54.79% |  |  |  |
| 2 or 3 | 34 | 39.08% |  |  | 33 | 45.21% |  |  |  |
| Surgery Type |  |  |  |  |  |  |  |  | *X^2^*(1, 160) = 1.3 |
| Lumpectomy | 62 | 71.26% |  |  | 45 | 61.64% |  |  |  |
| Mastectomy | 25 | 28.74% |  |  | 28 | 38.36% |  |  |  |
| Received chemotherapy |  |  |  |  |  |  |  |  | *X^2^*(1, 160) = .80 |
| Yes | 44 | 50.57% |  |  | 43 | 58.9% |  |  |  |
| No | 43 | 49.43% |  |  | 30 | 41.1% |  |  |  |
| Received radiation therapy |  |  |  |  |  |  |  |  | *X^2^*(1, 160) = .68 |
| Yes | 68 | 78.16% |  |  | 52 | 71.23% |  |  |  |
| No | 19 | 21.84% |  |  | 21 | 28.77% |  |  |  |
| Received endocrine therapy |  |  |  |  |  |  |  |  | *X^2^*(1, 160) < 0.01 |
| Yes | 64 | 73.56% |  |  | 55 | 75.34% |  |  |  |
| No | 23 | 26.44% |  |  | 18 | 24.66% |  |  |  |
| * denotes statistically significant comparison statistics (p < .05). ^a^Median Negative Affect = 15. | | | | | | | | | |

| **Table S9.**  Demographics and Clinical Characteristics of the Sample Compared on the Basis of T1 Sleep Disturbance. | | | | | | | | | |
| --- | --- | --- | --- | --- | --- | --- | --- | --- | --- |
|  | Low Sleep Disturbance  (≤ 5) | | | | High Sleep Disturbance  (> 5) | | | |  |
| Variable | N | % | Mean | (SD) | N | % | Mean | (SD) | Comparison Statistic |
| Age (years) | 50 |  | 51.52 | (8.32) | 107 |  | 51.66 | (8.14) | *t*(94) = .11 |
| Education |  |  |  |  |  |  |  |  | *X^2^*(2, 157) = .84 |
| Less than College | 10 | 20% |  |  | 22 | 20.56% |  |  |  |
| College Degree | 18 | 36% |  |  | 31 | 28.97% |  |  |  |
| Post-Graduate Degree | 22 | 44% |  |  | 54 | 50.47% |  |  |  |
| Race/Ethnicity |  |  |  |  |  |  |  |  | *X^2^*(4, 157) = 3.4 |
| White/Caucasian | 43 | 86% |  |  | 81 | 75.7% |  |  |  |
| Hispanic/Latino | 4 | 8% |  |  | 12 | 11.21% |  |  |  |
| Black/African American | 0 | 0% |  |  | 5 | 4.67% |  |  |  |
| Asian | 2 | 4% |  |  | 6 | 5.61% |  |  |  |
| Other | 1 | 2% |  |  | 3 | 2.8% |  |  |  |
| Married |  |  |  |  |  |  |  |  | *X^2^*(1, 157) < .01 |
| Married or living as married | 33 | 66% |  |  | 71 | 66.36% |  |  |  |
| Divorced, separated, widowed, or never married | 17 | 34% |  |  | 36 | 33.64% |  |  |  |
| Income |  |  |  |  |  |  |  |  | *X^2^*(1, 154) < .01 |
| < $100,000 | 16 | 33.33% |  |  | 36 | 33.96% |  |  |  |
| > $100,000 | 32 | 66.67% |  |  | 70 | 66.04% |  |  |  |
| Breast Cancer Stage |  |  |  |  |  |  |  |  | *X^2^*(1, 157) = 4.7* |
| 0 or 1 | 36 | 72% |  |  | 56 | 52.34% |  |  |  |
| 2 or 3 | 14 | 28% |  |  | 51 | 47.66% |  |  |  |
| Surgery Type |  |  |  |  |  |  |  |  | *X^2^*(1, 157) = 1.5 |
| Lumpectomy | 37 | 74% |  |  | 67 | 62.62% |  |  |  |
| Mastectomy | 13 | 26% |  |  | 40 | 37.38% |  |  |  |
| Received chemotherapy |  |  |  |  |  |  |  |  | *X^2^*(1, 157) = 10.1* |
| Yes | 17 | 34% |  |  | 67 | 62.62% |  |  |  |
| No | 33 | 66% |  |  | 40 | 37.38% |  |  |  |
| Received radiation therapy |  |  |  |  |  |  |  |  | *X^2^*(1, 157) < .01 |
| Yes | 38 | 76% |  |  | 79 | 73.83% |  |  |  |
| No | 12 | 24% |  |  | 28 | 26.17% |  |  |  |
| Received endocrine therapy |  |  |  |  |  |  |  |  | *X^2^*(1, 157) = 1.2 |
| Yes | 34 | 68% |  |  | 83 | 77.57% |  |  |  |
| No | 16 | 32% |  |  | 24 | 22.43% |  |  |  |
| * denotes statistically significant comparison statistics (p < .05). | | | | | | | | | |


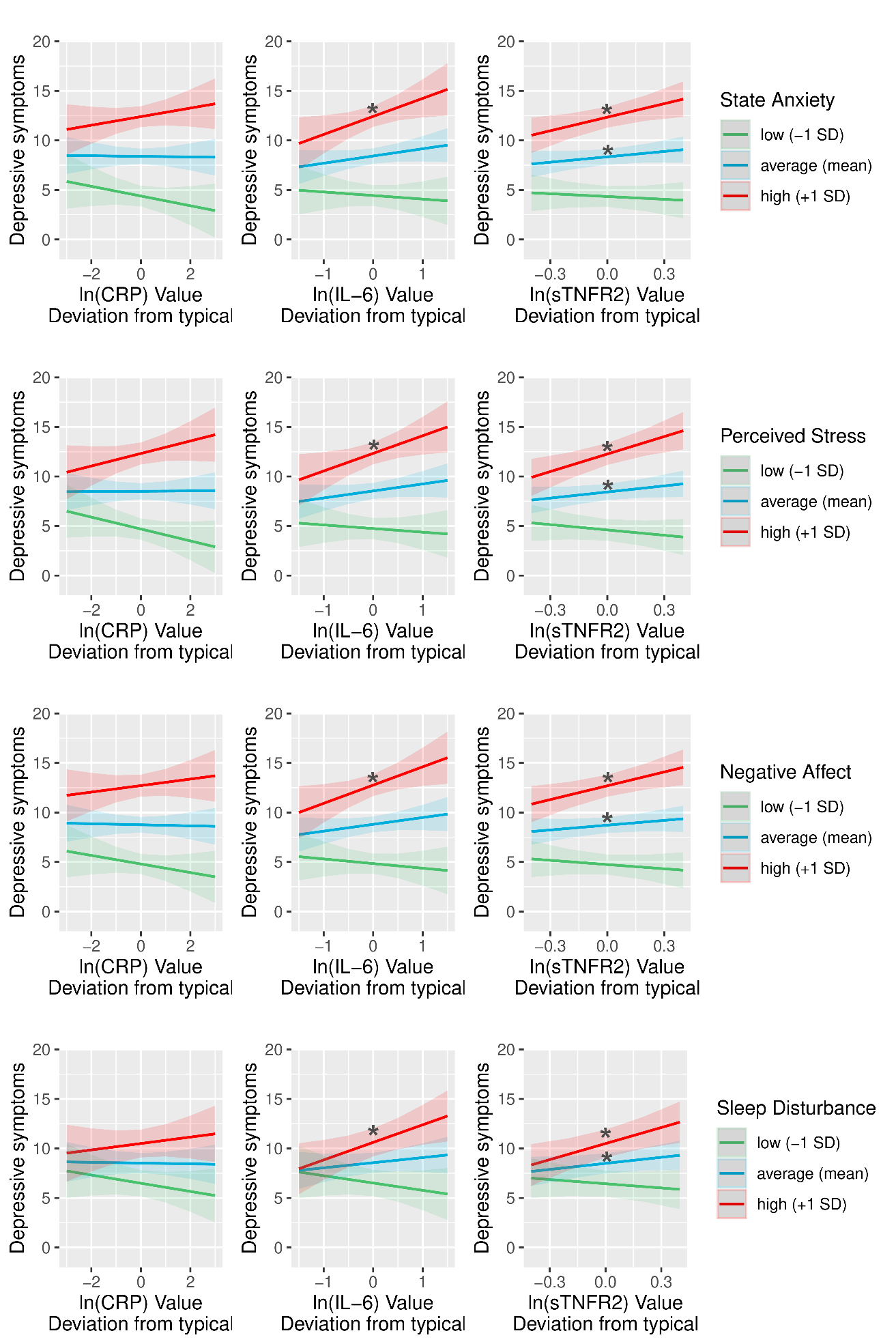


*Figure S1.* Predicted depressive symptom scores as a function of inflammation (CRP, IL-6, or sTNF-RII) and psychological risk factors (state anxiety, perceived stress, negative affect, and sleep disturbance). Inflammation (as indexed by IL-6 and sTNF-RII) was positively associated with depressive symptoms among women who reported high levels (+1 SD) of anxiety, perceived stress, negative affect and sleep disturbance. A similar (but non-significant) pattern of results emerged for CRP. Predicted depressive symptom scores were adjusted for age, BMI, race, surgery, cancer stage and receipt of chemotherapy, radiation therapy and endocrine therapy. Shaded areas depict confidence intervals of simple slopes. The asterisk symbols (*) index statistically significant simple slopes (*p* < .05).


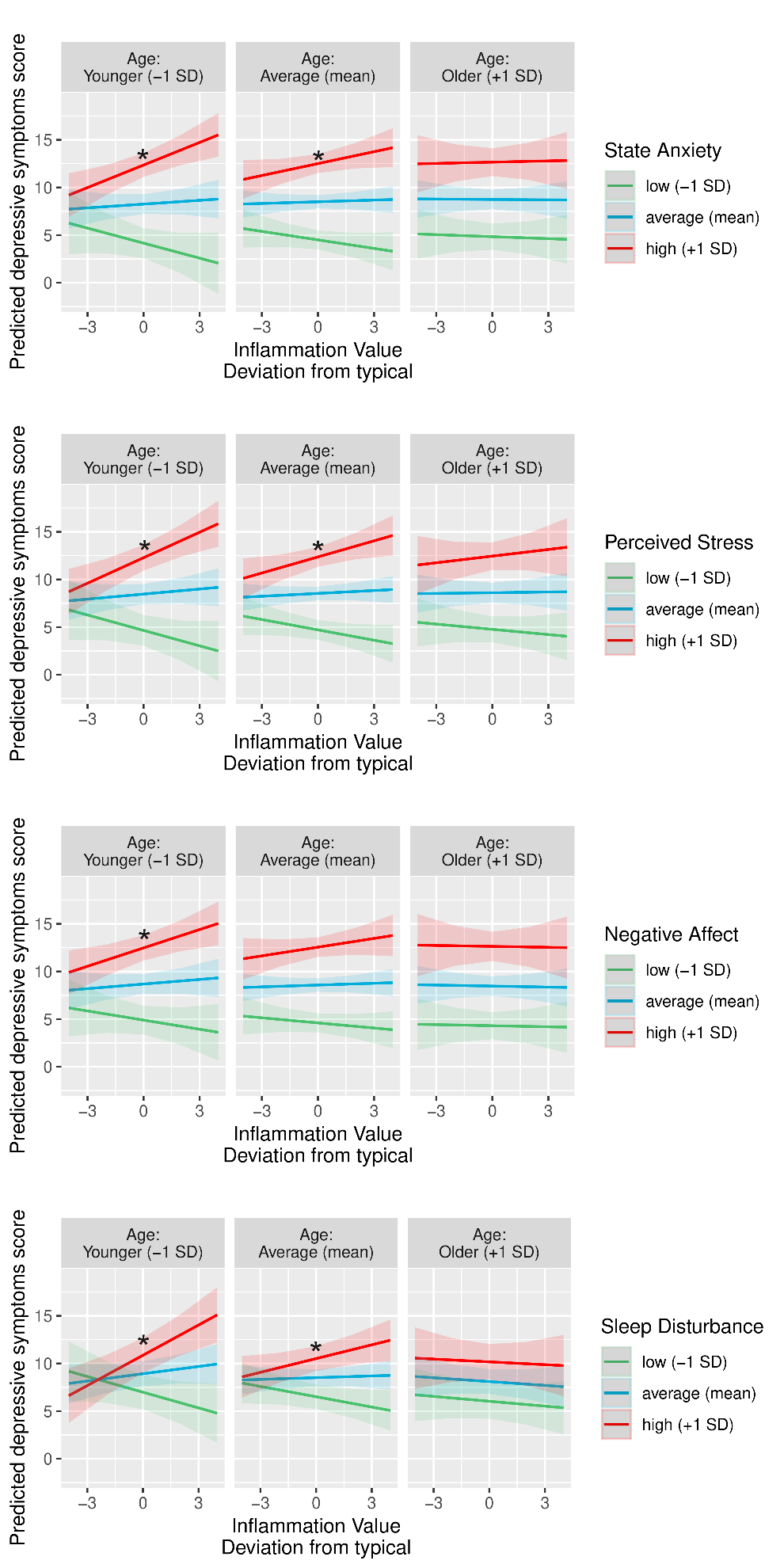


*Figure S2.* Predicted depressive symptom scores as a function of age, inflammation (scores on the composite inflammatory index), and key risk factors (state anxiety, perceived stress, negative affect, and sleep disturbance). Inflammation was positively associated with depressive symptoms among younger women who reported high levels (+1 SD) of anxiety, perceived stress, negative affect and sleep disturbance. Similarly, inflammation was positively associated with depressive symptoms among women of average age who reported high levels (+1 SD) of anxiety, perceived stress, and sleep disturbance. Predicted depressive symptom scores were adjusted for age, BMI, race, surgery, cancer stage and receipt of chemotherapy, radiation therapy and endocrine therapy. Shaded areas depict confidence intervals of simple slopes. The asterisk symbols (*) index statistically significant simple slopes (*p* < .05).
